# A review of pharmaceutical removal from wastewater via hydrodynamic cavitation

**DOI:** 10.1016/j.ultsonch.2026.107843

**Published:** 2026-04-01

**Authors:** Pengyun Liu, Emanuela Calcio Gaudino, Judy Lee, Giancarlo Cravotto

**Affiliations:** a Department of Drug Science and Technology, University of Turin, via P. Giuria 9, 10125 Turin, Italy; b School of Chemistry and Chemical and Process Engineering, University of Surrey, Guildford GU2 7XH, UK

**Keywords:** Pharmaceutical degradation, Wastewater treatment, Water remediation, Cavitational technology, Vortex techniques

## Abstract

Hydrodynamic cavitation (HC) is a powerful advanced oxidation process for water and wastewater remediation, but the degradation mechanism of pharmaceuticals in water matrices remains unclear. This comprehensive review aims to inspire and guide future research in this field by reviewing the removal of pharmaceuticals in water and wastewater using various HC hybrid processes, including standalone HC, and HC combined with H_2_O_2_, persulfate, peroxymonosulfate, percarbonate, Fenton, catalysts, ozonation, as well as HC/Photocatalysis. Given that HC/Plasma processes for water and wastewater remediation were comprehensively reviewed in our previous work, they are not discussed here. This review systematically examines the mechanisms, applications, and key influencing factors of these HC-based processes. In addition, the environmental impacts and sustainability aspects of HC technologies are highlighted. Finally, current challenges and future perspectives are presented to outline emerging issues and advanced research directions in this field.

## Graphical abstract

**Figure:**
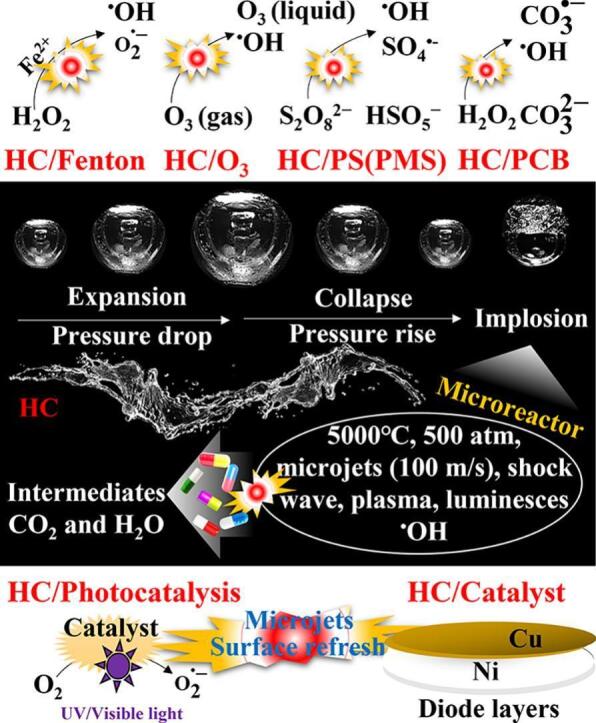

## 1 Introduction

**Fig. 1:**
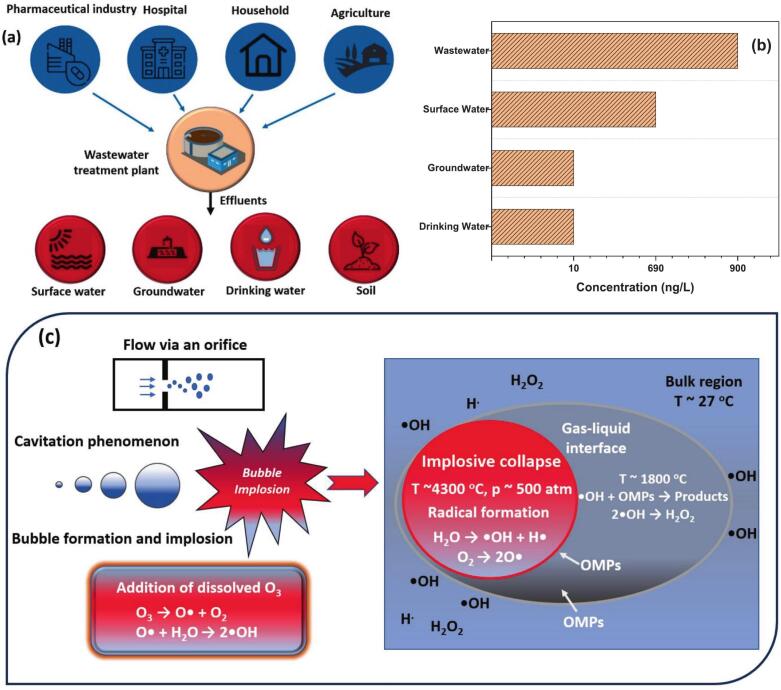
Sources of pharmaceuticals in environments (a); residue levels of pharmaceuticals in water matrices (b); mechanisms of HC (c). Reprinted from ref. [2] Copyright (2025), with permission from Elsevier. Note: OMPs refer to the organic micropollutants.

The extensive annual production and consumption of pharmaceuticals has contributed to the growing occurrence of pharmaceutical residues in wastewater matrices through a variety of transport pathways, as demonstrated in Fig. 1a [1], [2]. Pharmaceutical contaminants, like biological metabolites, endocrine disruptors, *β*-blockers, antibiotics, biguanide drugs, vitamins, and anti-inflammatory drugs, have been frequently detected in water and wastewater matrices from pharmaceutical industry effluents (up to very high ppm levels), municipal wastewater (up to ppm levels), livestock and poultry excrement, landfill leachate, groundwater, surface water (up to sub-ppm levels), river water (up to ppm levels), seawater, and drinking water, among others (Fig. 1b) [3], [4], [5], [6], [7], [8]. For instance, 0.50 × 10^-5^ – 0.53 × 10^-3^ ppm oseltamivir phosphate (OP) and 0.3 × 10^-4^ – 1.2 × 10^-3^ ppm oseltamivir carboxylate in sewage wastewater in Norway [9], 0.6 × 10^-4^ – 0.9 × 10^-3^ ppm oseltamivir carboxylate in river water in Japan [10], [11], 61–180 ppm tetracyclines (TCs) in pharmaceutical industry wastewater [12], and 0.06 and 0.33 ppm ceftriaxone in wastewater and groundwater, respectively [13], have been detected. As compiled in Table S1, pharmaceuticals are typically characterized by complex chemical structures with multiple bioactivities. As a result, they often exhibit biological resistance, recalcitrance, and toxicity in the environment, posing potential health risks to the aquatic ecosystems, human beings, animals, and microorganisms even at trace concentrations. Reported impacts include inhibition of ${\text{NH}}_{\text{4}}^{+}$-N oxidation and methane generation in water, disruption of the endocrine system, and the abnormal reproductive and developmental processes of aquatic organisms. These effects may ultimately lead to chronic diseases, including cancer, in humans, as well as growth inhibition, mutations, and the development of drug resistance in microorganisms [12], [14], [15], [16], [17], [18]. Therefore, the effective and rapid removal and mineralization of pharmaceuticals from water matrices (particularly wastewater, before discharge) to reduce the contaminant loading, total organic carbon (TOC), and chemical oxygen demand (COD) is imperative.

However, conventional physical, chemical, and biological approaches, like adsorption, precipitation, chlorination, as well as treatment by activated sludge, biofilm-reactor, and plant remediation, fail to effectively remove and mineralize pharmaceuticals simultaneously [19], [20], [21], [22], [23]. Advanced oxidation processes (AOPs) [24], such as photocatalysis [25], Fenton oxidation [26], sonochemical processes [7], [27], [28], [29], ozonation [30], [31], electrochemical oxidation [32], [33], and plasma processes [34], have attracted increased interest in water and wastewater treatment in recent decades due to the generation of powerful hydroxyl radical (^•^OH). These ^•^OH can non-selectively attack chemical molecules of pharmaceutical contaminants with reaction rates of 10^8^ to 10^10^ L/(mol·s) [35]. However, the main limitations of AOorifice plates becomes crucialPs are the production of undesired toxic intermediates (*e.g.*, nitro-containing compounds) and sludge, insufficient mineralization, and economic feasibility in large-scale applications [4], [15], [36], [37]. Our previous work has detailed the characteristics, merits, and limitations of conventional and common AOPs for water treatment [38]. Based on the types of pharmaceuticals, herein, the relevant typical approaches are compiled in Table 1.

**Table 1:** Typical processes for degrading pharmaceuticals in water [19], [20], [21], [22], [23], [25], [26], [32], [39].

Pharmaceuticals	Examples	Typical approaches	Mechanisms
Analgesics/anti-inflammatories	Ibuprofen, diclofenac, paracetamol	Ozonation/ Photocatalysis	High reactivity with ^•^OH due to electron-rich aromatic rings.
Antibiotics	Sulfamethoxazole, ciprofloxacin, amoxicillin	Fenton/ Electrochemical	Antibiotics are often resistant to biological treatment; AOPs break the stable rings that confer antimicrobial properties.
Antiepileptics	Carbamazepine	AOPs	Notoriously “recalcitrant” to biological systems; requires high-energy radicals to crack the molecule.
Hormones/steroids	Estradiol, ethinylestradiol	Adsorption/UV-AOP	Highly potent even at low levels; physical removal or UV-driven oxidation is very effective.
Lipid regulators	Clofibrate, bezafibrate	Biological/ Ozonation	Moderately biodegradable, but ozonation ensures mineralization of metabolites.
X-ray contrast Media	Iopromide, iohexol	Electrochemical /Plasma	Contain heavy iodine atoms; require high-energy processes to strip the halogens.

Cavitational processes have been well established and proven as effective advanced oxidation processes for water and wastewater remediation [40], [41], [42], [43]. These processes primarily rely on cavitation phenomena, which involve extreme physical and chemical effects induced by the rapid formation and violent implosion of ultrafine bubbles within microseconds in the liquid phase [44], [45]. As these bubbles implode, extremely high temperatures (1,000 – 10,000 K) and pressures (100 – 5,000 bar) are created within the bubble core, which serves as microreactors, producing microjets, shear force, turbulence, as well as the formation of ^•^OH due to the pyrolysis of water molecules [7], [27], [28], [29], [30], [46], [47], [48], [49], [50], [51], [52], [53]. Under such extreme conditions, pharmaceutical molecules are expected to be decomposed by radical attack, pyrolysis, and combustion in the presence of dissolved or injected O_2_ [54], [55].

Among various strategies for cavitation generation, piezoceramic transducers and hydraulic systems are frequently used for the degradation of contaminants in water and wastewater matrices, which are known as acoustic cavitation (AC) and hydrodynamic cavitation (HC), respectively [7], [56], [57]. Compared to AC, HC is able to instantly generate sufficient tiny bubbles with flexible configurations of the constriction units, induce high cavitation yields (*CYs*), and run with less energy consumption and lower costs [8], [58], [59]. In addition, it has been well known that HC systems are flexible to combine with other emerging techniques and easy to be scaled up, even on an industrial scale [60], [61], [62]. In general, scaling up HC processes requires balancing energy density (or cavitational activity) with mechanical durability. Although HC efficiently generates hydroxyl radicals to degrade complex drug molecules, the high-velocity micro-jets and shockwaves responsible for this degradation also cause significant surface erosion of reactor components, leading to frequent maintenance and the need for specialised, expensive materials. Additionally, as system capacity increases, managing pressure losses across Venturi or orifice plates becomes crucial; poorly optimised geometries can result in excessive energy dissipation without a corresponding increase in chemical oxidation. As a result, energy efficiency (typically measured by electrical energy per order) often fluctuates considerably at larger scales, as the power required to maintain the necessary discharge pressures can outweigh the degradation benefits unless the system is synergistically integrated with other treatments, such as UV, ozonation, or cold plasma, among others [60], [63], [64], [65], [66]. Typical demonstrations in this field are summarised in Table 2.

**Table 2:** Typical HC-dominant processes for pharmaceutical removal at pilot or industrial scales.

Scale	Processes	Pharmaceuticals	Core results	*Ref.*
Industrial Scale	HC/O_3_	Morphine, Thebaine (TCM wastewater)	Achieved 94% removal of morphine in 1 h and 99.9% removal of morphine and thebaine in 3 h under real-world conditions.	[67]
Pilot Scale (14 L)	Nanobubble-based HC	Humic acid, climbazole, sulfadiazine	Optimized Venturi geometry produced 90% nanobubbles (< 1 μm). Persulfate-assisted HC reached 56–88% degradation.	[68]
Pilot Scale (5 L)	HC/Cold Plasma	Furosemide	100% furosemide (50 mg/L) was removed in 10 min.	[69]
Pilot Scale	HC/H_2_O_2_/UV	Carbamazepine, diclofenac	Resulted in > 98% removal for carbamazepine and diclofenac.	[70]

HC on its own usually exhibits poor degradation efficiency (*DE*) and mineralization [31], [57], [58]. Although the higher *CYs* than AC, HC alone (10^-8^-10^-7^ mol/L/min) creates less ^•^OH than AC (10^-6^-10^-5^ mol/L/min) in liquid reaction systems than AC due to the production of low-quality (*e.g.*, high vapor levels) or ultra-stable bubbles (*e.g.*, super-cavitation bubbles), followed by fewer cavitation events as well as weaker cavitation intensity and effects [71], [72], [73], [74], [75], [76], [77], [78]. To enhance treatment efficiency, extensive efforts have been made to couple HC with various oxidants (*e.g.*, hydrogen peroxide (H_2_O_2_), persulfates (PS), peroxydisulfate (PDS), and percarbonate (PCB)), catalysts (mainly the metal coated layer over diodes), and AOPs (*e.g.*, Fenton or Fenton-like processes ozonation, photocatalysis, and plasma) [1], [23], [79], [80], [81]. The improved degradation observed in these hybrid processes generally arises from synergistic effects attributed to the enhanced production of reactive oxidative species (ROS, Reactions 1–48 in the Supplementary material), such as H_2_O_2_, ^•^OH, sulfate radicals (${\text{SO}}_{\text{4}}^{∙-}$), superoxide anion (${\text{O}}_{2}^{∙-}$), atomic oxygen (^•^O), and singlet oxygen (^1^O_2_), as well as improved mass transfer, and the intensified interactions between these oxidants and the target substrates in liquid phase [14].

The oxidation potentials of ^•^OH, ${\text{SO}}_{\text{4}}^{∙-}$, ${\text{NO}}_{\text{3}}^{∙-}$, ^•^O, O_3_, ${\text{HSO}}_{\text{5}}^{\text{-}}$, S_2_${\text{O}}_{\text{8}}^{\text{2-}}$, H_2_O_2_, ${\text{CO}}_{\text{3}}^{∙-}$, ^1^O_2_, and ${\text{O}}_{2}^{∙-}$ are 2.8, 2.6, 2.3–2.5, 2.42, 2.1, 2.1, 1.82, 1.78, 1.59, 1.07, and 0.33 eV, respectively [1], [2], [82]. In addition to the reaction rates between these oxidative species with pharmaceutical molecules, the distribution of these oxidative species with respect to the contaminant molecules around cavitation bubbles is also critical for achieving high degradation rates [28], [29], [30], [31], [48]. In general, hydrophobic pharmaceuticals tend to accumulate near the gas–liquid interface of cavitation bubbles, where high local ROS concentrations enhance degradation. In contrast, hydrophilic pharmaceuticals are less efficiently oxidized due to the limited diffusion of ^•^OH into the bulk liquid (∼10%). Volatile molecules inside bubbles can be degraded via both pyrolysis and oxidation by ^•^OH during cavitation [8], [28], [29], [31], [48]. Additionally, bubbles usually carry negative charges, which can attract pollutants with positive charges, thereby enhancing degradation performance [83]. Both HC alone and its hybrid processes have been applied for the removal of a wide range of contaminants in water and wastewater matrices. In addition to the improved *DE*, the hybrid HC processes also offer advantages of high energy effectiveness and low use of chemical reagents [56], [57], [80], [84].

Several reviews have focused on the application of the standalone HC processes in water and wastewater treatments [61], [84], [85], [86], [87], [88]. Therefore, this review will focus on both individual and hybrid HC systems, including HC/H_2_O_2_, HC/PS (or PMS), HC/PCB, HC/Fenton, HC/Ozonation (or HC/O_3_), and HC/Photocatalysis, for pharmaceutical removal in water and wastewater. For each process, the underlying mechanisms, applications, and the effect of key operational parameters are discussed. HC-plasma processes for the removal of pharmaceuticals have been comprehensively reviewed in our previous works [26] and are therefore not covered here.

## 2 Pharmaceutical removal by HC alone

### 2.1 Mechanism of HC

**Fig. 2:**
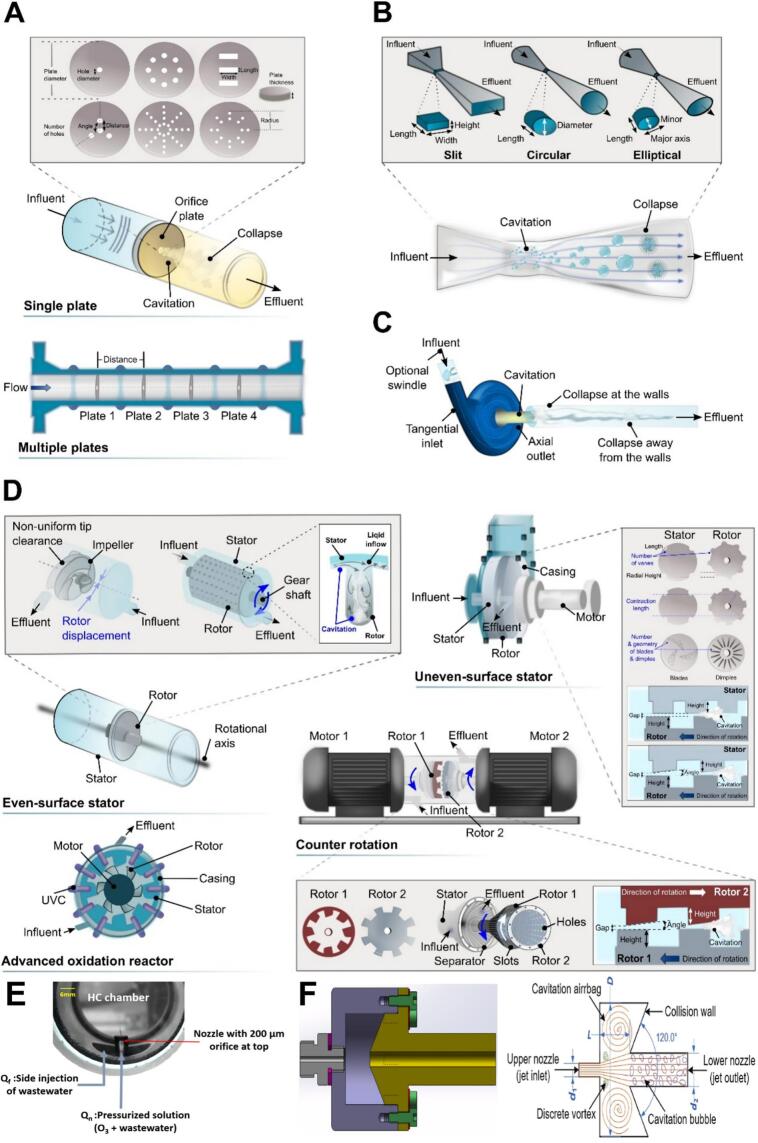
Throttling units: orifice plate (A), Venturi tube (B), vortex diode (C), rotational (D), nozzle (E), and self-excited oscillation cavity (F) [90]. Reprinted from ref. [2], [90], [91] Copyright (2025), with permission from Elsevier.

HC refers to the cavitation phenomenon induced by an instantaneous pressure drop (below the vapor pressure) in fluids, which can be triggered by hydraulic regulation using throttling units such as orifice plates [1], [83], Venturi tubes [89], vortex diodes [5], [22], rotor–stator assemblies [20], nozzles [3], self-excited oscillation cavity [90], and so forth [83] (Fig. 2).

The geometry of these throttling units is imperative to ensure better cavitation and degradation [92]. The orifice plates and Venturi tubes can be installed at the suction and extrusion sides, while the rotor (located at the impeller or drive shaft) and stator (located at the pump cavity or inner wall) are positioned relatively. The installation of vortex diodes depends on the specific application. Reactors designed based on the rotor–stator assembly are quite complex and energy demanding, while the orifice-based reactors have the simplest design [4], [20], [78], [93]. Bhandari *et al.* highlighted that the vortex diode-based reactors offer advantages such as swirling flow, smaller pressure drop (∼0.48 bar versus ∼1.4 bar in linear flow-based reactors), faster cavitation inception and nucleation, larger chambers, greater opening areas, lower choking, higher cost-effectiveness, and less energy consumption [4], [15], [21], [94]. Among them, the orifice plate-based and venturi-based reactors are most frequently applied. Venturi tubes generally produce larger and more numerous bubbles, while the orifice plates are simpler, flexible, and cost-effective [4].

In HC, cavitation is promoted not only by localized pressure drops but also by the intense and instant reduction of flow regions and increase in flow velocity. Under comparable inlet pressure (*P_i_*), venturi units generate higher throat velocity than the orifice plate counterpart owing to the smooth geometric construction [95]. The size and density of the generated bubbles depend on fluid conditions, influencing the cavitation characteristics, including the number of HC events and the intensity of HC [54], [96]. Higher *P_i_* values (or flow velocity) generally favor the occurrence of cavitation, but excessively high *P_i_* values (or flow velocity) may trigger the formation of large stable cavitation clouds (also known as super-cavitation phenomena). Optimal *P_i_* values (or flow velocity) exist for HC systems, depending on the geometry of the constriction units and the operating conditions [14], [96].

The cavitation number (*C_v_*) is another key parameter for evaluating cavitation efficiency and predicting degradation performance. Higher input pressure and flow velocities can decrease *C_v_* (≤1) which corresponds to more intense cavitation, more ROS generation, and thus better *DEs* [97]. Too low *C_v_* values can reduce cavitation intensity due to the occurrence of super-cavitation. Optimal performance is often observed when *C_v_* values are in the range from 0.6 to 1.0 [15]. In addition, *CYs* (*i.e.*, the degradation achieved per unit of power consumed), energy efficiency, and treatment cost are also crucial for evaluating the feasibility of HC processes and comparing different HC systems [4], [5].

In HC systems, feed liquids can be circulated continuously through the throttling units and when combined with the enhanced mass transfer and mixing, this allows contaminants decomposition, although mineralization is typically low and *DEs* per pass are often below 40% [43], [54], [82], [92]. The primary merits of individual HC include no sludge formation or secondary pollution, ease of operation and maintenance, environmental friendliness, low costs, robustness, broad applicability, and excellent scalability [18], [22].

### 2.2 Application of HC alone for pharmaceutical removal

The typical applications of HC alone for the degradation of various pharmaceuticals in water and waste matrices are shown in Table 3.

**Table 3:** The degradations of various pharmaceuticals in water and waste matrices by HC alone.

Pollutants	Matrix	Conditions	*DEs* (%)	*CYs* (mg/J)	Energy efficiency or total cost	Other results	*Refs.*
Cephalexin (CFX)	Aqueous solution	Orifice plate-based reactor, 2 mm × 5 holes, pH 5, *C_v_* of 0.162, 1 h	24	*−*	*−*	*DE* of TOC was 17%.	[96]
CFX	Aqueous solution	Orifice plate-based reactor, 5 ppm, 2 bar, 2 h	−	−	−	*DE* of TOC was 60%	[98]
Chlortetracycline	Distilled water	Venturi-based reactor, 2 bar, 5 min	50	−	−	−	[13]
Ciprofloxacin	Demineralized water	Skid-mounted rotating, 400 L (pilot scale), 1 h	45	0.1––0.3	1.03 to 1.05 kWh/m^3^ (Rs. 5.27/m^3^)	*DEs* of TOC and COD were 7% and 60% in 0.5 h, respectively	[20]
Neomycin	Aqueous solution	Orifice plate-based reactor, pH 3, 5 bar, 1 h	55	2.2 × 10^−4^		*−*	[44]
Sulfadiazine	Millipore water	Orifice plate-based reactor, 4 × 2 mm holes, 20 ppm	8		6034 kWh/m^3^	*−*	[2]
Oseltamivir phosphate	Distilled water	Venturi-based reactor, pH 10, 5 bar, 1.5 h		6.3 × 10^−5^	0.7 × 10^−3^ USD/(L.mg)	*DE* of TOC was 64% with a *k* value of 15 × 10^−3^ min^−1^	[4]
Prazosinhydrochloride	Synthetic wastewater	Diode-based reactor, 20 L, 10 mg/L, pH 4, 0.5 bar, 1 h	15–30	*−*	*−*	*Max DE* of TOC was 35%	[21]
Pharmaceuticals	Industrial effluents	Orifice-based reactor, 10 L, *C_v_* < 0.7, 1.5 h	−	−	−	*Min.* pH change. *Max. DE* of BOD, COD, and TDS were 20%, 6%, and ∼ 14%, respectively	[15]
		Venturi tube-based reactor, 8 mm of throat diameter, same other conditions	−	−	−	*Min.* pH change. *Max. DE* of TDS was 8%	
Pefloxacin	Deionized water	Orifice plate-based reactor, 10 mg/L, pH 3.3, 3 bar, 2 h	85	−	−	−	[24]
Sulfamerazine	Milli-Q water	Venturi-based reactor, circular (HC_CV_) Venturi tube, 5 L, 4 bar, pH 5.6	22	4.1 × 10^−4^	−	Energy consumptions were ordered as HC_SV_ > HC_DSV_ > HC_CV_	[99]
		Slit (HC_SV_) Venturi tube, same other conditions	28	2.2 × 10^−4^			
		Dual-slit Venturi (HC_DSV_) Venturi tube, same other conditions	42	3.7 × 10^−^^4^			
Tetracycline	Aqueous solution	Venturi-based reactor, 30 mg/L, 25℃, 0.35 bar, 1 h	20	−	−	*−*	[100]

Note: *DEs*, degradation efficiencies; *CYs*, cavitation yields; *Refs*, references.

**Fig. 3:**
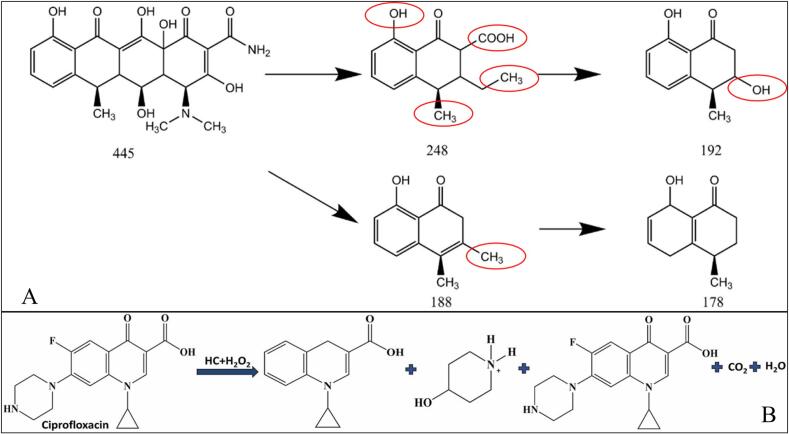
Presumed DOX degradation pathways by HC alone (A). Reprinted from ref. [92] Copyright (2025), with permission from Elsevier; CIP degradation pathway by HC/H_2_O_2_ (B). Reprinted from ref. [17] Copyright (2022), with permission from Elsevier.

As shown in Table 3, HC generally leads to limited *DEs* and insufficient mineralization of various pharmaceuticals. Agarkoti *et al.* [98] reported that electricity consumption increased with the throat area of venturi tubes and volumetric flow velocity. Similarly, Mukherjee *et al.* observed that increasing the rotational speed of the skid-mounted rotating increased the energy consumption, with an optimal *CY* value identified for CIP degradation at a pilot scale [19]. A typical example is illustrated in Fig. 3A showing DOX degradation by HC alone.

### 2.3 Roles of effective factors

#### 2.3.1 Effect of the type of constriction units

Various constriction units have been employed in HC systems, but results regarding the most effective design remain controversial. For example, Warade *et al.* [14] reported that the orifice unit is more effective for the treatment of industrial pharmaceutical effluents by HC alone compared to the conventional meter and Venturi meters. They stated that in Venturi-based HC systems, HC-induced radicals significantly decreased effluent pH, whereas this effect was not observed in the orifice design due to the development of the orifice *vena contracta*. The high *DEs* of the orifice-based reactors are attributed to the optimal *C_v_* value (<0.7) and the intense cavitation effects [15]. Conversely, Katiyar *et al.* found that Venturi units achieved superior removal of OP (69%) compared to orifice plates (48%), owing to the smooth inner surface of the constriction unit, higher pressure reduction, faster flow velocity, and more sustained cavitation with longer bubble lifetimes and low energy loss. In contrast, the sudden pressure drop in the orifice unit can limit the evolution of bubbles, followed by weak cavitation effects and low exposure of OP to cavitation [4].

#### 2.3.2 Effect of the design of venturi tubes

The half divergent angles (HDAs) influence cavitation by altering fluid velocity and pressure recovery, thereby affecting the degradation performance [101]. Wu *et al.* degraded doxycycline (DOX) by HC alone using Venturi tubes with HDAs of 5°, 6°, 7°, 8°, and 9° for 2 h, and corresponding *DEs* of DOX were 42%, 52%, 40%, 38%, and 35% under the conditions of 5 L, pH 5.6, and 4 bar. Lower HDA (5°) causes slower flow velocity (5.05 m/s), longer cavitation zone, and higher cavitation intensity, while higher HDAs increased flow velocity (9.80 – 12.32 m/s) and accelerated recovery in the divergent region, which reduced the bubble residence time and weakened cavitation effects [92]. The higher HDA of HC_DSV_ (6.5°) than HC_CV_ (6.4°) and HC_SV_ (5.5°), resulting in faster pressure recovery and more intense cavitation with using HC_DSV_ [99].

Similarly, the half-convergent angles (HCAs) can also affect the cavitation performance. Using venturi-based reactors with half convergent angles of 21°, 22°, 23°, 24°, and 25° for DOX degradation, the corresponding *DEs* were 46%, 52%, 49%, 47%, and 47%, respectively. The highest *DE* at 22° is attributed to the large cavitation area, long bubble lifetime, and high turbulent kinetic energy. While large HCAs can enhance cavitation, excessively high HCAs can reduce cavitation intensity due to increased flow resistance caused by the collision and friction among H_2_O molecules in the throat of venturi tubes [92], [102].

The height/length ratio (H/L) of the venturi throat determines the local retention time of the bubbles with very short or long retention times limiting bubble development and consolidation [103]. Agarkoti *et al.* reported that a higher H/L value of HC_CV_ (1.0) enhances the degradation compared to the HC_SV_ (0.95) and HC_DSV_ (0.5) [98].

The height/width ratio (H/W) of the Venturi throat controls the length of cavitation zones, where higher W/H values create larger inlet edges and improved shear zone, intensifying cavitation[103]. For example, HC_DSV_ with a W/H value of 4.00 led to better *DEs* than HC_SV_ with a W/H of 3.16 [99].

The throat perimeter/cross-sectional area ratio (*α*) determines the cavitation bubble density. Usually, high *α* values will produce more bubbles, larger shear zones, and thus enhanced cavitation effects [3], [104]. Agarkoti *et al.* evaluated the degradation of SMZ at α values of 2 (HC_CV_), 1.38 (HC_SV_), and 2.5 (HC_DSV_), with corresponding *C_v_* values of 0.167, 0.275, and 0.227, and the number of passes of 78, 190, and 170. These configurations resulted in *DEs* of 21.54%, 27.69%, and 41.54%, respectively [98].

#### 2.3.3 Effect of the design of orifice plates

The design of orifice plates strongly influences cavitation in HC systems. The number of holes affects the effective cavitation area: fewer holes create smaller flow areas and higher flow constriction which enhances cavitation. Conversely, more holes reduce cavitation intensity due to the collision, extrusion, and shorter bubble residence time [54], [96]. For example, Devia-Orjuela *et al.* reported that orifices with 5 holes achieved higher *DEs* and mineralization of CFX than those with 7 holes, due to lower *C_v_* values and more intense cavitation [96]. The diameter of the orifice distribution zone (*D_X_*) determines controls the spacing between the orifices, and thus cavitation bubble dynamics. Low *D_X_* values result in insufficient distances among orifices, causing collision and extrusion among cavitation bubbles, reducing their residence. However, excessively high *D_X_* values can increase local head loss and weaken cavitation effects[55]. The length of the orifice plate inlet zone (*D_Y_*) affects the local turbulence intensity at the orifice plate, which in turn influences cavitation [105]. Similarly, if the outlet zone length (*D_Z_*) is too short, it will limit the cavitation zone, and if too-long it will reduce the interaction between ROS and the target substrates [55]. A typical case by Yi *et al.* confirmed the optimal *D_X_*, *D_Y_*, and *D_Z_* values were 32, 100, and 200 mm for CTC removal by HC alone in the ranges of 19–40, 100–300, and 100–300 mm, respectively [55].

#### 2.3.4 Effect of the design of self-excited oscillatory HC reactors

The length of the oscillation chamber plays a critical role in maximizing the oscillatory effect in HC reactors. An overly short chamber will limit the cavitation bubble development, whilst if the chamber is too long, it will cause pulse energy dissipation and weaken pulse jets, resulting in low cavitation intensity [106]. Wang *et al.* investigated the degradation of TC using a self-excited oscillatory HC reactor, and after 2 h of treatment, decreasing chamber length of 23, 20, 25, and 18 mm led to a decrease in *DEs* of 51% (with a *k* value of 5.8 × 10^−3^ min^−1^), 40%, 42%, and 33%, respectively [82].

The inlet to outlet diameter ratio affects the flow characteristics, such as flow rate and the turbulent intensity. Enhanced Turbulence improves the bubble mixing and collision, thereby favoring degradation. At high ratios (>1), jet flow is limited to the outlet side, with suppression backflow, which stabilizes pressure within the oscillation chamber and inhibits the occurrence of pulse jets. In contrast, low ratios will improve the flow rate and pulsation impacts, resulting in stronger cavitation events [106]. Wang *et al.* reported that an inlet to outlet ratio of 0.7 led to higher *DEs* of TC than ratios of 1.9 and 1.7, with a degradation rate of 5.8 × 10 ^−3^ min ^−1^ [82].

#### 2.3.5 Effect of the rotational speed of the rotor

Appropriate rotor speed improves bubble generation and cavitation intensity. Although excessive speed can induce more bubble production, it can decrease the energy released during cavitation and induce slip between the rotor and water, thus limiting cavitation intensity and degradation [107], [108]. Mukherjee *et al.* showed that by increasing the rotor’s rotational speed from 1,500 to 2,700  rpm, 1 h treatment of CIP by HC alone resulted in an increase in *DEs* from 20% to 45%, and *k* values from 3.1 × 10^-3^ to 8.5 × 10^-3^ min^−1^[19].

#### 2.3.6 Effect of inlet pressure

**Fig. 4:**
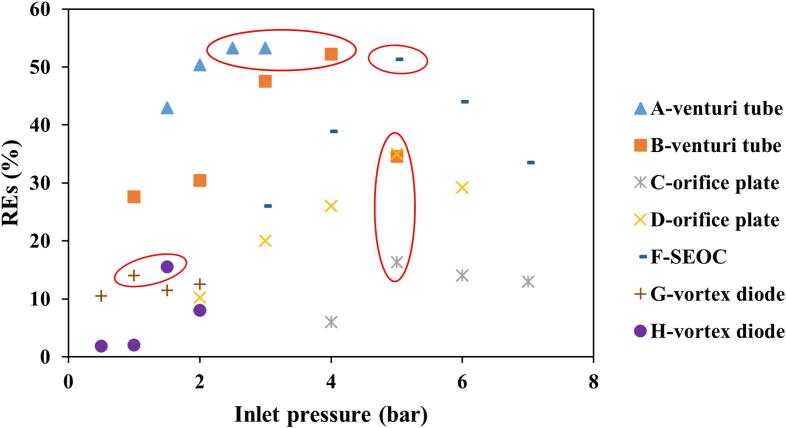
Effect of inlet pressure on *REs* of various pharmaceuticals by HC alone. A: CTC [13]; B: DOX [92]; C: TOC [16]; D: NEM [44]; E: PEF [24]; F: TC [82]; G: TOC [5]; H: TOC [16]. Note: SEOC refers to the self-excited oscillating cavitation.

Optimal *P_i_* values depend on the reactor design and physicochemical properties of pharmaceutical contaminants [21] and the underlying degradation mechanisms associated with *P_i_* or *C_v_* have been discussed in Section 2.1. In brief, low *P_i_* values are insufficient to initiate effective cavitation, while excessively high *P_i_* values will weaken the cavitation effects due to the occurrence of super-cavitation. Generally, there exists an optimum *P_i_* value for maximum *DE*. Fig. 4 demonstrates the effect of inlet pressure on *REs* of various pharmaceuticals by HC alone using different types of reactors.

As shown, the presence of an optimal *P_i_* value is confirmed, regardless of HC reactor type. As red circled in Fig. 4, the optimal *P_i_* values for Venturi-, orifice-, SEOC-, and vortex diode-based reactors are 2.5–4.0 bar, 5 bar, 5 bar, and 1.0–1.5 bar, respectively. Remarkably, vortex diode units are able to create the best cavitation conditions at very low *P_i_* values compared to the other throttling units. A case by Patil *et al.* [16] described that the optimal *P_i_* value was 1.5 and 5.0 bar for orifice- and vortex diode-based reactors, respectively, to reach similar *DEs* of TOC during CIP removal by HC alone [16].

#### 2.3.7 Effect of reaction pH

Reaction pH affects the degradation of pharmaceuticals via changing their chemical speciation (*i.e.*, molecules, positive and negative charged states) in water matrices, especially for diode-based HC processes [4]. For instance, the CFX molecule exists in the cationic form at low pH (<2.56), anionic form at high pH (>6.88), and in the molecular form at pH 2.56–6.88 due to its –NH_3_ and –COOH functional groups [96]. Metformin exhibits similar pH dependency due to its p*K*a values of 2.8 and 11.5 [5]. The charge of pharmaceutical molecules in liquids affects their location with respect to the cavitation bubbles, resulting in different degradation efficiencies [8], [28], [29], [31], [48]. It has been reported that molecular pharmaceuticals (p*K*a > reaction pH) are more prone to ^•^OH oxidation and pyrolysis [20]. Table S1 presents the p*K*a values of common pharmaceuticals. Acidic conditions often enhance degradation by promoting more ^•^OH formation via Reactions 3 and 9 under high oxidative potential and inhibiting ^•^OH recombination due to the suppressive effect of H^+^ [3]. However, strongly acidic conditions can corrode the metal components, reducing the lifetime of HC reactors [24]. Despite high pH can also limit the ^•^OH recombination, it will decrease the oxidative ability of ^•^OH to 1.5 eV [3], [96]. The effect of pH on the degradation of various pharmaceuticals by HC alone is shown in Table 4.

**Table 4:** The effect of pH on various pharmaceutical degradation by HC alone.

Throttling units	Pollutants	Optimal pH	Highest *DEs* (%)	pH effects	*Ref.*
Orifice plate	Cephalexin	5.0	−	Negligible effect using 5 × holes orifice, while increasing pH (5–7) reduced *DEs*	[96]
	Chlortetracycline	3.0	81	Alkaline conditions were not conducive to degradation	[55]
	Neomycin	3.0	55	Lowering the pH from 7 to 3 increased *DEs*	[44]
	Pefloxacin	3.3	85	Decreasing the pH from 10 to 3.3 increased *DEs*	[24]
	Oseltamivir phosphate	10.0	48	Decreasing the pH from 10 to 2 reduced *DEs*	[4]
Rotor-Stator	Ciprofloxacin	2.0	45	Decreasing the pH from 10 to 2 increased *DEs*	[20]
Vortex diode	Metformin	2.0	100	Increasing the pH from 2 to 6 reduced the *DEs*	[5]

Note: *DEs*, degradation efficiencies; *Refs*, references.

As shown in Table 4, in most cases, acid conditions (pH 2–5) are conducive to the pharmaceutical removal. Patil *et al.* stated that the pH adjustment is especially crucial in vortex diode-based reactors [5].

#### 2.3.8 Effect of initial concentration

In general, low initial pharmaceutical concentration favors higher *DEs* because ^•^OH concentrations and cavitation intensities remain relatively constant regardless of the pharmaceutical concentrations, and there is less competition between the pollutant and intermediates with ROS. Although higher concentrations often result in low *DEs*, a greater absolute number of molecules can be degraded under the same operating conditions [17], [44].

The majority of researchers confirmed that high initial pharmaceutical concentrations led to poor degradation [16], [17], [24], [44]. Whilst contrasting behaviors were reported by Yi *et al.* using HC alone, where increasing CTC loading from 5 to 15 mg/L resulted in an increase in *DEs* from 60% to 81%. The author attributed the higher *DEs* to the improved trapping of CTC by ^•^OH at higher loading [55]. Patil *et al.* reported higher *DEs* and *CYs* of MIT at 20 mg/L (5% and 162 × 10^–5^ mg/J) compared to 10 mg/L (3% and 96 × 10^–5^ mg/J) [5]. Similarly, another study also reported an increase in *DEs* of CIP as the concentration is increased from 10 to 100 mg/L, *DE* increased from 21% (TOC reduction: 6%) to 56% (TOC reduction: 16%), respectively, and the relevant *CYs* were 1.9 × 10^−4^ and 48.5 × 10^−4^ mg/J [17]. In addition, Wang *et al.* increased TC concentration from 5 to 20 mg/L and found a maximum *DE* (51%) and *k* (3.2 × 10^−3^ min^−1^) at 10 mg/L. The author indicated that the low *DE* at a low initial concentration of 5 mg/L was due to the insufficient interaction between TC and cavitation bubbles [82].

Overall, *CY* values are crucial for determining the optimal reaction concentration in the practical implementation of HC processes. For example, low concentrations are usually favorable if high *DEs* are desired, whilst appropriately increasing concentrations can shorten the treatment period while maintaining high *DEs* due to the improved *CY* [24] and reduce the treatment costs [17].

#### 2.3.9 Effect of residence time

Long operation time facilitates the degradation of pollutants by increasing the cumulative ROS concentration, while excessively long treatment times may increase the energy consumption with marginal gains in *DEs* [89]. In contrast, short treatment times enable smaller reactor designs and improve the cost-effectiveness, and reduce the footprint [96].

It was reported that increasing treatment times from 0.5 and 1.0 h in a HC system (pH 5 and 5 holes in the cavitation plate) increased the *DEs* of CFX (and mineralization rates) from 22% (16%) to 24% (17%) [96]. Mukherjee *et al.* investigated the effect of retention time (corresponding to different numbers of passes) on the degradation of CIP by rotor-based HC alone at 2,700 rpm for 1 h, and the *DEs* were 25% and 45% at passes of 90 and 120, respectively. Insufficient number of passes will result in low flow velocity and static pressure, thereby limiting cavitation occurrence and bubble residence time. Whereas, excessive numbers of passes can suppress cavitation due to the poor coupling between the rotor and the water layer, and low pressure drop [19].

#### 2.3.10 Effect of aeration

Air can be introduced into HC systems through bubbling or injection at the suction side, or self-injected under low pressure. Aeration can enhance cavitation intensity and ROS generation by promoting bubble nucleation and thus increasing degradation. However, the extent of this improvement is contaminants dependent and excessive aeration can induce physical carryover of bubbles, which can negatively affect the cavitation activities [17].

Patil *et al.* found *DEs* of TOC during the degradation of 10 and 20 mg/L MTF increased by 233% and 200% by HC with air bubbling compared to individual HC, respectively [5]. However, another work reported that the aeration in HC systems decreased the *DEs* of 10 and 100 mg/L CIP by 71% and 57%, respectively [17]. Moreover, Dixit *et al.* reported that *DEs* of TOC during the degradation of 10 ppm active pharmaceutical ingredients (API) by HC at 1.5 bar with and without aeration were 17% and 16%, respectively, demonstrating that no synergistic effects occurred [15].

Hence, the aeration should be used with caution to avoid possible inhibition impacts.

#### 2.3.11 Effect of co-existing ions

Co-existing ions can rapidly react with ^•^OH through Reactions 49–67 (See Supplementary materials) in HC systems to form low-oxidative species and inhibit degradation [43], [54], [95], [109]. Moreover, some anions can affect the biological and chemical properties of pharmaceuticals via complexation [82]. In addition, metal cations can decrease the saturated vapor pressure of water, hindering the bubble formation and leading to fewer cavitation events and lower cavitation intensity [55].

Yi *et al.* investigated the impact of Cl^−^, ${\text{CO}}_{\text{3}}^{\text{2-}}$, and ${\text{SO}}_{\text{4}}^{\text{2-}}$ on the degradation of CTC by HC alone and found that 0.5 h treatment resulted in a decrease in *DE* from 59% (without ions) to 9% (Cl^−^), 51% (${\text{CO}}_{\text{3}}^{\text{2-}}$) and 48% (${\text{SO}}_{\text{4}}^{\text{2-}}$). Further increase in treatment time to 1 h resulted in *DE* of 79% (without ions), which decreased in the presence of ions to 59% (Cl^−^), 63% (${\text{CO}}_{\text{3}}^{\text{2-}}$) and 59% (${\text{SO}}_{\text{4}}^{\text{2-}}$). Similarly, the presence of Na^+^, Mg^2+^, and Ca^2+^ reduced *DEs* of CTC by HC alone from 59% to 49%, 42% and 37% at 0.5 h and from 79% to 59%, 65% and 50% in 1.0 h, under the same conditions [55]. Wang *et al.* also reported a similar decrease in the degradation of TC for 2 h by HC alone from 51% to 29%, 44%, and 31% in the presence of ${\text{CO}}_{\text{3}}^{\text{2-}}$, ${\text{NO}}_{\text{3}}^{\text{-}}$ and ${\text{SO}}_{\text{4}}^{\text{2-}}$, respectively [82]. In another study, Wang *et al.* reported a higher degradation of TC by HC alone in ultrapure water (51%) compared to tap water (31%), *Qinhu Lake* water (22%), and *Huangjia Lake* water (26%). The author attributed this reduced degradation to the co-existing ions in the real water matrix [82], which inhibited the degradation through Reactions 49, 52, 64, and 66. These results indicate that pretreatment to remove these anions and cations from water matrices will be beneficial prior to HC application. Nevertheless, this can be impractical and costly. To our knowledge, alternative strategies encompass higher oxidant dose, staged processes, hybrid-biological trains, and so forth, may also favor, which are comparatively assessed in Table 5.

**Table 5:** Pretreatment strategies of pharmaceutical wastewater before HC treatments.

Option	Primary mechanism	Pros	Cons
Chemical pre-treatment	Removes scavengers (Cl^−^, ${\text{HCO}}_{\text{3}}^{\text{-}}$) via ion exchange/softening.	Maximizes HC efficiency; prevents scale formation in equipment.	Very high cost; generates a secondary waste stream (brine/sludge).
Higher oxidant dose	Increases radical concentration to overcome scavenging.	Very low infrastructure cost; easy to automate.	High OPEX (chemical costs); potential for quench effect from the oxidant itself.
Staged processes	Sequential treatment zones to optimize reaction kinetics.	Better utilization of energy; prevents “hot spots” of chemical concentration.	Increased footprint; more complex piping and control systems.
Hybrid-bio trains	HC increases “BOD/COD” ratio for biological finishing.	Lowest overall cost for high-volume wastewater; most sustainable.	Requires careful control to ensure HC effluent isn't toxic to microbes.
Filtration/ Centrifugation	Physical removal of total suspended solids.	Protects HC nozzles from erosion and clogging.	Requires frequent backwashing or filter replacement.
pH Adjustment	Chemical dosing to reach optimal radical generation range.	Low cost; can convert carbonates into CO_2_.	Requires constant monitoring; adds salt content to effluent.
Coagulation/ Flocculation	Aggregating colloids and metals for removal.	Reduces “background” load for more targeted HC.	Large footprint for settling tanks; high sludge volume.
Flotation	Using air bubbles to lift oils, grease, and light solids to the surface.	Highly effective for removing hydrophobic scavengers and fats.	High energy for air compression; requires skimming mechanisms.
Acoustic/Sound Flotation	Ultrasonic waves aggregate particles and micro-bubbles for removal.	No chemicals needed; highly effective on fine/colloidal particles.	High power consumption; expensive to scale for high flow rates.
Adsorption (bulk and column)	Pollutants/ions stick to the surface of activated carbon.	Can target specific inhibitory ions or large organic competitors.	High cost for carbon regeneration or replacement; can clog easily.
Pre-Ozonation	Initial chemical cracking of long-chain molecules.	Strong synergy; O_3_ enhances cavitation intensity.	High electricity cost for ozone generation.

#### 2.3.12 Effect of scavengers

Scavengers favor the identification of radicals and their roles in HC systems. For instance, methanol and *tert*-butanol can be used to determine ^•^OH, while p-benzoquinone can be used to detect ${\text{O}}_{2}^{∙-}$ [82]. Wang *et al.* found that *DEs* of TC by HC alone in the presence of methanol, *tert*-butanol, and p-benzoquinone for 2 h were 26%, 35%, and 19%, respectively, identifying the dominant roles of ^•^OH and ${\text{O}}_{2}^{∙-}$ during TC degradation [82].

## 3 Pharmaceutical removal by HC/Oxidants

Oxidants like H_2_O_2_, S_2_ ${\text{O}}_{\text{8}}^{\text{2-}}$, ${\text{HSO}}_{\text{5}}^{\text{-}}$, and Na_2_CO_3_·1.5H_2_O_2_ can be activated by HC to improve the performance of pharmaceutical removal.

### 3.1 Pharmaceutical removal by HC/H_2_O_2_

#### 3.1.1 Mechanism of HC/H_2_O_2_

Although H_2_O_2_ and HC alone lead to slow degradation of pharmaceuticals, combining H_2_O_2_ with HC systems can enhance the formation of ^•^OH via Reactions 3, 9, and 12–14 under cavitation-induced extreme conditions, thereby improving degradation [44]. However, too much H_2_O_2_ will consume ^•^OH to form ${\text{HO}}_{2}^{∙}$, and ${\text{O}}_{2}^{∙-}$ with low oxidative potential via Reactions 4, 10, and 11, which limits the degradation efficiency [4], [15], [43], [95]. The optimal dosage of H_2_O_2_ depends on the specific properties of the target pharmaceuticals [5].

#### 3.1.2 Application of HC/H_2_O_2_ for pharmaceutical removal

The typical examples of HC/H_2_O_2_ for pharmaceutical removal are compiled in Table 6.

**Table 6:** The application of HC/H_2_O_2_ for pharmaceutical degradation in water matrices.

Pollutants	Optimal conditions	*DEs* (%)	*k* values (×10^−3^ min^−1^)	Synergistic index (*SI*)	Other results	*Ref.*
Active pharmaceutical ingredient (API)	Al-diode based reactor, 20 L, 10 mg/L, molar ratio API: H_2_O_2_ of 1:200, 0.5 bar, 0.17 h	100	−	57.56	−	[21]
Chlortetracycline (CTC)	Orifice-based reactor, 80 ppm CTC, 8 mmol H_2_O_2_, pH 7, 3 bar, 0.08 h	89	−	−	*DE* in 0.5 h was 94%	[55]
	10 mg/L CTC, same other conditions	95	−	−	*DE* by HC alone was 79%. Mineralization rate reached 61% in 1 h	
Chlortetracycline	Venturi-based reactor, 1 L, 80 ppm, 8 mM H_2_O_2_, pH 6, 25°C, 2 bar, 0.5 h	94	−	−	*CY* was 9.5 × 10^−5^ mg/J	[13]
Ciprofloxacin (CIP)	Diode-based reactor, 10 ppm CIP, mole ratio CIP/H_2_O_2_ of 1:750, 1.1 g/L H_2_O_2_, 1.5 bar,	32	2.3	1.89	*DEs* of TOC by single processes are 0.6%-15.5%. *CY*s were (15–23.4) × 10^−5^ mg/J	[16]
	Aeration and a diode-based reactor, same other conditions	34	3	2.11		
	Orifice-based reactor, 5 bar, 10 ppm CIP, mole ratio CIP/H_2_O_2_ of 1:750, 1.1 g/L H_2_O_2_	21	2	1.18	*DEs* of TOC by single processes were 0.6%-16.3%. *CY*s were (3.7–4.5) × 10^−5^ mg/J	
	Aeration and orifice-based reactor, same other conditions	25	2	1.62		
CIP	Rotor-based HC reactor, aqueous solution, 0.3 g/L H_2_O_2_, 0.5 h	86	62	8.13	*DE* of TOC was 30%. Adding H_2_O_2_ improved *CY* and energy consumption by 1.93 and 1.88 times, respectively	[20]
CIP	Diode-based reactor, 10 ppm CIP, molar ratio of CIP: H_2_O_2_ of 1:1000, 3 h	80		2.80	*CY* was 7.2 × 10^−4^ mg/J	[17]
	100 ppm CIP, same other conditions	95		2.60	*CY* was 86.8 × 10^−4^ mg/J. Higher CIP concentration reduced treatment costs	
Metformin (MET)	Diode-based reactor, 1 m^3^/h, 10 ppm MET, mole ratio MET/H_2_O_2_ of 1:500, pH 6.3–6.8, 1 bar 3 h	10	1	2.00	*DEs* by single processes were 3%-5%. *CY*s were (101–166) × 10^–5^ by HC/H_2_O_2_. pH was crucial	[5]
	20 ppm MET, mole ratio MTF/H_2_O_2_ of 1:700, same other conditions	33	2	4.20		
	10 ppm MET, molar ratio MET/H_2_O_2_ of 1:500, same other conditions	100	49	2.80		
	20 ppm MET, mole ratio MET/H_2_O_2_ of 1:700, same other conditions	88	13	11.3		
Neomycin (NEM)	Orifice-based reactor, 35 mM, molar H_2_O_2_/NEM ratio of 321:1, pH 3, 5 bar, 0.42 h	80	−	2.03	Energy efficiency was 7.9 × 10^−4^ mg/J. *Max. DE* by H_2_O_2_ alone was 72% in 1 h.	[44]
Naproxen carbamazepine, diclofenac	Venturi-based reactor, 1 L, 0.001 ppm, 20 mL 30% H_2_O_2_, 6 bar, 0.5 h	86, 72, 77, respectively	−	−	*−*	[110]
Oseltamivir phosphate (OP)	Orifice-based reactor, molar ratio of OP: H_2_O_2_ of 1:40 μΜ, 10 ppm OP, pH 10, 5 bar, 1.5 h	100	55	2.75	*DEs* of OP and TOC by HC alone were 22%, and 20%, respectively. *DE* of TOC was 79%	[4]
Pefloxacin (PEF)	Orifice-based reactor, molar ratio of PEF: H_2_O_2_ of 1:5, pH 5.3, 3 bar	70	19	4.40	*DE* by HC alone was 30%.	[24]
	Pharmaceutical wastewater, 400 mL, same other conditions	44	−	−	−	
Sulfamerazine	Venturi-based reactor, HC_DSV_ (or HC_SV_, and HC_CV_), 5 L, 0.7 mM H_2_O_2_, pH 5.6, 4 bar	63 (or 49 and 44)			−	[99]

Note: *DEs*, degradation efficiencies; *k*, reaction rate constants; *Refs*, references.

As listed in Table 6, adding H_2_O_2_ in the HC system significantly enhanced the pharmaceutical degradation, often exhibiting strong synergistic effects, particularly with pH variation. The optimal conditions depend on the pharmaceuticals. Mukherjee *et al.* reported that the inhibition efficiency of Chlorella vulgaris’s growth was up to 90% after 96  h of incubation (toxicity of 5.7%), while the toxicity gradually reduced with prolonged treatment for each incubation stage [20]. HC/H_2_O_2_ exhibited higher *CY*s than HC alone, especially with pH change. *CYs* depend on the initial concentration, reaction pH, type of reactors, and HC systems. The diode-based systems exhibited higher energy efficiency and cost-effectiveness compared to orifice-based systems (Table 6) [5], [17]. Katiyar *et al.* stated that the treatment cost was decreased by 8.8-fold during the OP degradation by HC/H_2_O_2_ (0.67 USD/L/g) compared to HC alone [4], while Patil *et al.* found that adding H_2_O_2_ increased treatment costs [5]. A typical example of CIP degradation by HC/H_2_O_2_ is shown in Fig. 3B.

#### 3.1.3 Roles of effective factors

##### 3.1.3.1 Effect of reaction pH

Reaction pH can affect the ^•^OH production derived from H_2_O_2_. At extremely low pH, ^•^OH production is limited by the accumulation of H^+^ near H_2_O_2_. At high pH, H_2_O_2_ mainly decomposes to H_2_O and O_2_, and the produced ^•^OH readily recombines. Hence, high pH reduces degradation efficiency due to the limited ^•^OH production [44].

Patil *et al.* reported that adding H_2_O_2_ and adjusting pH to 4 achieved complete MET removal (10 mg/L and 1 bar for 3 h), increasing *DE* by 900% compared to HC alone. However, this synergistic effect was reduced when the pH was increased to 8 [5].

##### 3.1.3.2 Effect of the H_2_O_2_ dosage and initial concentration

The Mechanisms governing the effect of H_2_O_2_ dosage are described in Section 3.1.1. In HC/H_2_O_2_ processes, *DE* generally increases with increasing H_2_O_2_ dosage up to an optimal value beyond which further addition can reduce *DEs*.

Yi *et al.* found that the optimal molar CTC to H_2_O_2_ ratio was 1.00:0.75 within the investigated range of 1.0:0.5–1:1, resulting in *DEs* of 70% and 95% after 0.5 and 1 h of treatment, respectively [55]. Similarly, the optimal molar contaminant/H_2_O_2_ ratios were 1:40, 1:700, 1:750, and 1:1,000 for the degradation of CIP [20], OP [4], CTC [13], MET [5], naproxen [16], and CIP [17], respectively (Table 6). Additionally, Liu *et al.* found that *DEs* of 30 mg/L TC by HC/UV/H_2_O_2_ increased with increasing H_2_O_2_ dosage from 0.5 to 2.0 mM, and the highest *DE* was 91% [100]. Patil *et al.* stated that CIP was mainly mineralized by HC alone at low concentration (10  mg/L), while higher *DEs* of CIP and TOC were observed at 100 mg/L by HC/H_2_O_2_. Meanwhile, both the higher *DEs* of CIP and TOC were obtained at 100 mg/L than at 10 mg/L in HC/ H_2_O_2_ systems [17].

##### 3.1.3.3 Effect of aeration

Dixit *et al.* [15] reported that the synergistic index was 2.1 in HC/H_2_O_2_/Aeration systems for NAP degradation under optimal conditions, and aeration enhanced the degradation by > 100% compared to HC alone and HC/H_2_O_2_ [16]. Nevertheless, aeration is not always favorable, depending on the properties of pharmaceuticals and the reaction conditions. For example, Patil *et al*. stated an adverse result during MET removal [5].

### 3.2 Pharmaceutical removal by HC/PS

#### 3.2.1 Mechanism of HC/PS

Persulfate (PS) can be activated via HC to produce powerful ^•^OH and ${\text{SO}}_{\text{4}}^{∙-}$ through Reactions 23–24 [28], [30], [47], [83]. Radical ${\text{SO}}_{\text{4}}^{∙-}$ has merits of pH persistence, long lifespan, non-selective oxidation, and high oxidative ability and reaction stoichiometric efficiency [[13], [17], [99]]. Thus, pharmaceuticals and their intermediates can be decomposed by both ^•^OH and ${\text{SO}}_{\text{4}}^{∙-}$, leading to better degradation and mineralization.

#### 3.2.2 Application of HC/PS for pharmaceutical removal

The typical applications of HC/PS for pharmaceutical degradation are summarized in Table 7.

**Table 7:** Applications of HC/PS, HC/PMS, and HC/PCB for pharmaceutical degradation.

Pollutants	Optimal conditions	*DEs* (%)	Other results	*Ref.*
Atenolol (ATL)	75 mg/L PS, 15 ppm ATL, pH 4, 1.25 h.	65	*−*	[9]
Estrone (E1)	0.1 mM PS, 4.5 m^3^/h, 60°C, 4 s	99	*DEs* with and without heating were similar	[18]
E1	80 mg/L PCB, 0.3 μg/L E1, 4 s	97	Energy consumption was 2.2 kWh/m^3^/order. Temperature has negligible effects, and prolonged oxidation occurred	[77]
Sulfamerazine	0.92 mM PS, HC_CV_/PS, HC_SV_/PS, and HC_DSV_/PS, 5 L, pH 5.6, 4 bar	74, 84, and 88, respectively	HC_DSV_ is more suitable for PS and PMS activation	[99]
	0.83 mM PMS, same other conditions	88, 94, and 95, respectively		

Note: *DEs*, degradation efficiencies; *Refs*, references.

**Fig. 5:**
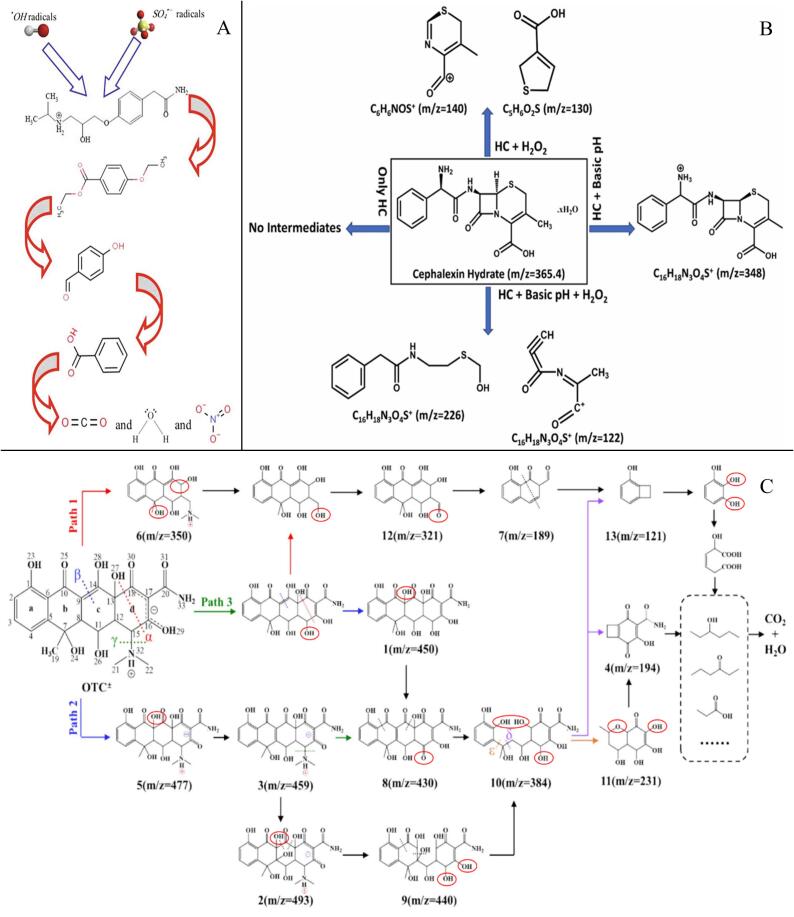
Presumed ATL degradation by HC/PS (A). Reprinted from ref. [9] Copyright (2021), with permission from Elsevier; Presumed CFX degradation by various Cu diode-based HC processes (B). Reprinted from ref. [94]. Copyright (2023), with permission from Elsevier; Presumed OTC degradation pathways by HC/O_3_ (C). Reprinted from ref. [23] Copyright (2025), with permission from Elsevier.

As shown in Table 7, the presence of additives can improve the pharmaceutical degradation. It has been reported that HC activation reduced energy consumption by 10-fold in comparison with AC [111]. Přibilová *et al.* reported that the energy consumption for degrading various Estrones using HC/PS (0.1 mM) in 4 s was 1.1–2.2 kWh/m^3^/order [18]. A typical example in Fig. 5A shows the ATL degradation by HC/PS.

#### 3.2.3 Roles of effective factors

##### 3.2.3.1 Degradation by various processes

Xiang *et al.* reported that adding 0.1 mM PS in HC systems increased *DEs* of 20 µM TC by 16- and 10-fold compared to individual HC and PS, respectively, under optimal pH 3.5, 2.4 bar, and 30°C for 2.5 h [83]. The corresponding *k* values for HC/PS, HC, and PS systems were 6.2 × 10^−3^, 3.8 × 10^−4^, and 6.1 × 10^−4^ min^−1^, resulting in a high SI value of 6.3 [83]. Similarly, Khajeh *et al.* found that the *DEs* and *k* values of 15 mg/L ATL at pH 4 for 1.25 h were < 1%, 37%, and 64.5%, and 1.1 × 10^−4^, 6.6 × 10^−3^, and 1.5 × 10^−2^ min^−1^ for individual PS and HC, and HC/PS, respectively [8].

##### 3.2.3.2 Effect of HC conditions

Xiang *et al.* [83] reported that *DEs* of TC treated for 2 h by HC/PS using orifices with circular, square, and triangular holes were 50%, 44%, and 34%, respectively. They attributed the higher *DE* with the circular hole to a larger cavitation area, stronger cavitation intensity, and better activation of PS. They also found that *DEs* of TC by HC/PS increased from 38% to 57% as *P_i_* values increased from 0.150 to 2.4 bar, likely due to the enhanced cavitation effects at higher pressures [83].

##### 3.2.3.3 Effect of solution pH, temperature, and initial concentration

At low pH, PS usually possesses fewer negative charges, while HC cavities have the identical charges, thus accelerating PS reactions and inducing more powerful degradation by ${\text{SO}}_{\text{4}}^{∙-}$. However, very low pH can limit the degradation by hindering the ${\text{SO}}_{\text{4}}^{∙-}$ production and promoting the transformation through Reactions 24–26. At higher pH, ^•^OH becomes the dominant radical due to the faster production through Reactions 24–27. Moreover, the much longer lifespan of ${\text{SO}}_{\text{4}}^{∙-}$ compared to ^•^OH also contributes to more effective degradation under acidic conditions[9]. In turn, the generated sulfates may also slightly reduce the reaction pH [18]. On the other hand, reaction temperature affects bubble formation, collapse dynamics, and the interactions with pharmaceutical pollutants. An optimal temperature promotes efficient ^•^OH formation, whereas high vapor pressure inside cavities will weaken the cavitation effects [3].

Khajeh *et al.* [8] reported that pH 6 (65%) induced higher *DEs* of 15 mg/L ATL by HC/PS than pH 3 (39%) and 9 (27%). They also found that at pH 6 and 10 min treatment by HC/PS, increasing ATL concentration from 10 to 60 and 75 mg/L decreased the *DEs* to 42% and 64%, respectively. This was attributed to the insufficient ${\text{SO}}_{\text{4}}^{∙-}$ and ^•^OH radicals at high ATL loading [8]. Xiang *et al.* stated that low pH favors TC degradation by HC/PS, with the highest DEs of 57% and 18% observed at pH 3.5 and 8.2, respectively. They also found that *DEs* of TC by HC/PS at 25, 30, and 35°C were ∼ 35%, 57%, and 51%, respectively, where temperature mainly changed the nature of cavitation bubbles (*e.g.*, amounts, size, and vapor content) instead of the cavitation volume [83]. Thus, appropriate pH, temperature, and initial concentration favor the pharmaceutical degradation by HC/PS.

##### 3.2.3.4 Effect on PS dosage

Similar to Section 3.1.3.2, optimal PS dosages also exist. Khajeh *et al.* reported that the degradation of 15 mg/L ATL by HC/PS at pH 6 increased at higher PS dosage, with *DEs* of 67% and 100% at 75 and 200 mg/L, respectively [8]. Přibilová *et al.* stated that 0.05 mM PS was sufficient for E1 removal, and increasing the dosage to 0.1 mM did not produce effects comparable to H_2_O_2_. They also noted that PS concentrations did not lead to significant differences in HC/PS systems with and without heating [18]. Xiang *et al.* reported that *DEs* of TC by HC/PS at TC/PS molar ratios of 1:1, 1:5, 1:10, and 1:20 were 19%, 57%, 60%, and 57%, respectively [83]. Therefore, optimizing PS dosage is crucial, and both insufficient and excessive amounts should be avoided.

##### 3.2.3.5 Effect on co-existing anions and scavengers

Khajeh *et al.* [8] found that treating 15 mg/L ATL by HC/PS in the presence of 15 mM ${\text{SO}}_{\text{4}}^{\text{2-}}$, I^−^, ${\text{NO}}_{\text{3}}^{\text{-}}$, ${\text{PO}}_{\text{4}}^{3\text{-}}$, and Cl^−^ at pH 6 for 100 min resulted in a *DE* of 99%, 98%, 94%, 91%, and 70%, respectively, which corresponds to *SI* values of 4.6, 3.6, 3.4, 2.2, and 1.1. The authors attributed the enhancement to the increased in ionic strength and the formation of anion-derived radicals through Reactions 52, 62, and 68–70 (See Supplementary materials). However, ${\text{CO}}_{\text{3}}^{2\text{-}}$ and ${\text{HCO}}_{\text{3}}^{\text{-}}$ inhibited the degradation, leading to *DEs* of 34% and 41%, respectively. This can be due to the consumption of ${\text{SO}}_{\text{4}}^{∙-}$ and ^•^OH by ${\text{CO}}_{\text{3}}^{2\text{-}}$ and ${\text{HCO}}_{\text{3}}^{\text{-}}$ through Reactions 49, 50, and 75, as well as the transformation of ${\text{CO}}_{\text{3}}^{∙-}$ through Reactions 71–72. They also found *DE* in tap water to be 1.07 times lower than in distilled water [9]. Xiang *et al.* found co-existing anions inhibited TC degradation, and *DEs* reduced from 57% to 25%, 43%, 37%, and 23%, in the presence of 2.5 mM Cl^−^, ${\text{NO}}_{\text{3}}^{\text{-}}$, ${\text{SO}}_{\text{4}}^{\text{2-}}$, and ${\text{HCO}}_{\text{3}}^{\text{-}}$, respectively. In addition to the reaction with ^•^OH through the above-mentioned reactions, these anions can also consume ${\text{SO}}_{\text{4}}^{∙-}$ through Reactions 73–75. Furthermore, the high-concentration ${\text{SO}}_{\text{4}}^{\text{2-}}$ can reduce the oxidative ability of ${\text{SO}}_{\text{4}}^{∙-}$ based on the Nernst formula [83]. Moreover, Aseev *et al.* reported that the inhibition efficiency of anions on the CEF degradation by HC/PS followed the order ${\text{HCO}}_{\text{3}}^{\text{-}}$ ≫ ${\text{SO}}_{\text{4}}^{\text{2-}}$ > Cl^−^ [14].

Scavengers can inhibit the pharmaceutical degradation by prioritizing the consumption of ${\text{SO}}_{\text{4}}^{∙-}$ and ^•^OH [9]. Khajeh *et al.* [8] reported that *DEs* of ATL by HC/PS (200 mM) without and with 15 mM *t*-butanol and 30 mg/L humic acid were 100%, 75%, and 54%, respectively, at optimal conditions. They found the contribution was 58% for ${\text{SO}}_{\text{4}}^{∙-}$ and 42% for ^•^OH in HC/PS systems [9]. Xiang *et al*. reported that the *DEs* of TC without and with the addition of 10 mM *t*-butanol and methanol by HC/PS were 57%, 17%, and 30%, respectively. And the contribution was 25% for ${\text{SO}}_{\text{4}}^{∙-}$ and 75% for ^•^OH in HC/PS systems [83]. Thus, the dominant radical is case dependent in HC/PS systems.

### 3.3 Pharmaceutical removal by HC/PMS

#### 3.3.1 Mechanism of HC/PMS

Likewise, PMS can also be activated by HC to form ${\text{SO}}_{\text{4}}^{∙-}$ and ^•^OH through Reaction 76, facilitating the pharmaceutical removal [89]. However, PS was reported to be more economical and cleaner than PMS in real-world implementation [112].

#### 3.3.2 Application of HC/PMS for pharmaceutical removal

The typical applications of HC/PMS for pharmaceutical degradation are shown in Table 7.

#### 3.3.3 Roles of the effective factors

##### 3.3.3.1 Degradation by various processes

Noori *et al.* reported that the *DEs* of 3 mg/L CBZ after 1.5 h at 2 mM PMS, pH 3, and 4.3 bar were 5% for UVC alone, 25% for HC alone, 30% for alone, 31% for UVC/PMS, 40% for HC/PMS, 67% for HC/UVC, and 73% for HC/PMS/UVC [89].

##### 3.3.3.2 Effect of pH

PMS is stable under acidic conditions, and ${\text{SO}}_{\text{4}}^{∙-}$ was reported to be the dominant radical in this case [99]. Noori *et al.* found that the optimal pH for CBZ removal was 5. At pH > 5, ${\text{SO}}_{\text{4}}^{∙-}$ and ^•^OH can be consumed through Reaction 77–78. At pH < 5, ${\text{SO}}_{\text{4}}^{∙-}$ will self-combine through Reactions 26 [89].

##### 3.3.3.3 Effect of PMS dosage

Increasing PMS dosage from 0.5 to 2.0 mM in HC systems under optimal conditions improved *DEs* of CBZ by ∼ 230%, leading to the *DE* up to 40% [89].

##### 3.3.3.4 Effect of initial concentration

Under optimum conditions, a higher initial concentration of CBZ reduced the *DEs* by HC/PMS with *DEs* at 3 and 10 mg/L being 41% and 73%, respectively [89]. These results are consistent with part of Section 2.3.8.

### 3.4 Pharmaceutical removal by HC/PCB

#### 3.4.1 Mechanism of HC/PCB

Compared to H_2_O_2_, PCB can derive extra oxidative radicals through Reactions 17 and 79–83 (see Supplementary materials), and offers additional merits of high cost-effectiveness, stability, safety, cleanliness, and stronger pH adaptability due to the buffer action of ${\text{CO}}_{\text{3}}^{\text{2-}}$ and ${\text{HCO}}_{\text{3}}^{\text{-}}$ [77].

#### 3.4.2 Application of HC/PCB for pharmaceutical removal

The typical applications of HC/PMS for pharmaceutical degradation are shown in Table 7.

#### 3.4.3 Roles of effective factors

##### 3.4.3.1 Effect of SPC dosage

Odehnalová *et al.* reported that the *DEs* of E1 in distilled water by HC/PCB for 4 s at PCB concentrations of 0.08 and 0.2 g/L were 31% and 47% (3.4 min^−1^), respectively, and the relevant *DEs* and *k* values for 20 s were 62% and 90% (7.3 min^−1^) [77].

## 4 Pharmaceutical removal by HC/Fenton and HC/Fenton-like processes

### 4.1 Mechanism of HC/Fenton and HC/Fenton-like

Generally, Fenton reagents are composed of H_2_O_2_ and Fe^2+^. In Fenton processes, Fe^2+^ acts as an initiator and catalyst for the generation of ^•^OH and ${\text{HO}}_{2}^{∙}$ from H_2_O_2_ through Reactions 84–85. Similarly, Reactions 87–89 (see Supplementary materials) in Fenton-like processes can also improve radical production. Usually, high Fe^2+^ concentration favors pharmaceutical degradation due to improved radical production. But excessive Fe^2+^ can consume ^•^OH through Reaction 86 and precipitate Fe^3+^ [99]. HC can decompose the iron complexes (which usually occur when pH > 4), promoting the regeneration of Fe^2+^ and H_2_O_2_ activation, followed by improved ^•^OH formation and better degradation. In addition, decreased pH to acid conditions and O_2_ production when adding Fenton or Fenton-like reagents has been widely reported, creating a better reaction environment for pharmaceutical degradation. The main drawbacks of HC/Fenton processes are sludge production, pH dependence, harmful chemical consumption, and possible secondary pollution by Fe^2+^ [1], [13], [18], [43], [82].

### 4.2 Application of HC/Fenton for pharmaceutical removal

The typical applications of HC/Fenton and HC/Fenton-like processes for pharmaceutical degradation are listed in Table 8.

**Table 8:** The applications of HC/Fenton and HC/Fenton-like processes for pharmaceutical degradation.

Pollutants	Optimal conditions	*DEs* (%)	*SI* values	Other results	*Ref.*
Ceftiofur (CEF)	Molar PS/CEF ratio of 10:1, 5 bar, 4 h	81	−	−	[14]
Chlortetracycline	1 L, 80 mg/L, pH 6, 25°C, 0.08 h	88	−	−	[13]
Ciprofloxacin	0.1 g/L FeSO_4_, 0.3 g/L H_2_O_2_, aqueous solution, 0.5 h	88	8.22	*DE* of TOC was 35% CY, energy efficiency, and total cost were 5.7 × 10^-4^ μg/J, 0.56 kWh/m^3^, Rs. 2.8/m^3^, respectively	[20]
	400 L wastewater, same other conditions	45	−	*−*	
Neomycin (NEM)	Molar Fe^2+^:H_2_O_2_ ratio of 0.15:1.00, 0.1 g/L NEM, pH 3, 27°C, 5 bar, 0.42 h	88	2.38	Energy efficiency was 8.7 × 10^−4^ mg/J *DE* by Fenton for 1 h was 80%	[44]
Oseltamivir phosphate	Molar FeSO_4_·7H_2_O/H_2_O_2_ ratio of 1:3, 10 μM H_2_O_2_, 1 L, 1.5 h	87	1.24	*DEs* of OP and TOC by Fenton alone were 12% and 11%, respectivelyTreatment cost was 0.00067 USD/ (L.mg)	[4]
Sulfadiazine (SDZ)	182 mg/L *α*-Fe_2_O_3_, 349 mg/L Na_2_S_2_O_8_, 0.95 mL/L H_2_O_2_, 20 ppm SDZ, 8.1 bar, pH 4	93	−	Energy efficiency was 194 kWh/m^3^/order	[2]
Sulfamerazine	0.75 mM Fe^2+^, 0.7 mM H_2_O_2_, 5 L, HC_DSV_/Fe^2+^/H_2_O_2_	97	−	Total cost was 0.18 ₹/L *CY* was 8.56 × 10^-4^	[99]
	5 L, 0.75 mM Fe^2+^, 0.7 mM H_2_O_2_, 22 + 44 kHz, UC/Fe^2+^/H_2_O_2_	97	−	Total cost was 0.46 ₹/L. CY was 3.24 × 10^-4^	
Tetracycline (TC)	Molar TC: Fe^2+^: H_2_O_2_ ratio of 1:1:10, 10 ppm TC, 5 bar, 2 h	86	1.73	*DE* by HC alone was 51%	[82]

Note: *DEs*, degradation efficiencies; *SI*, synergistic index; *Refs*, references.

As listed, HC/Fenton and HC/Fenton-like processes led to *DEs* of various pharmaceuticals over 80% with high *SI* values under optimal conditions. Gawande *et al.* stated that the energy efficiency of HC alone was 4-fold lower compared to the individual H_2_O_2_ and Fenton processes. The energy efficiency of HC/Fenton was slightly lower than those of individual H_2_O_2_ and Fenton processes, while HC/Fenton led to 4-fold faster degradation [44].

### 4.3 Roles of effective factors

#### 4.3.1 Degradation by various processes

The excellent ability of HC/Fenton processes over individual HC and Fenton processes can be confirmed by the high *SI* values in Table 8. Mukherjee *et al.* found that HC/Fenton processes (88%) also induced higher *DEs* of CIP than individual H_2_O_2_ (3%) and O_3_ processes (13%) [20]. Even the advantages of HC/Fenton processes compared to AC/Fenton or AC/Fenton-like processes for degrading pharmaceuticals have also been frequently reported, while the HC/AC/Fenton combinations appear more powerful [[12], [13], [99]].

#### 4.3.2 Effect of constriction units

Agarkoti *et al.* [98] reported that *DEs* of SMZ by HC_CV_/Fe^2+^/H_2_O_2_, HC_SV_/Fe^2+^/H_2_O_2_, and HC_DSV_/Fe^2+^/H_2_O_2_ under optimal 0.75 mM Fe^2+^, 0.74 mM Fe^3+^, 0.7  mM H_2_O_2_, 4  bar, 5  L, and pH 5.6 were 93%, 95%, and 97%, respectively, and the relevant *DEs* using Fe^3+^ instead of Fe^2+^ were 89%, 90%, and 94% [99]. Roy *et al.* stated that *DEs* of SDZ by HC/Fenton-like processes increased with increasing *α* and decreasing *β_0_* (refers to the total open area of the throttling units divided by the pipe's full cross-sectional area) values from 0.073 to 0.036, owing to the intensified cavitation intensities by the decreased flow area and turbulent degree, respectively. *k* values at 10 bar at α values of 1 (*β_0_* of 0.073) and 2 mm^−1^ (*β_0_* of 0.036) were 0.0414 and 0.0434 min^−1^, respectively. They also reported that orifice plates with smaller holes yielded better *DEs* of 20 ppm SDZ than those with larger holes. *DEs* by HC/PS/*α*-Fe_2_O_3_ using orifices with hole diameters of 2, 3, and 4 mm at 10 bar, pH 4, *α*-Fe_2_O_3_ 182 mg/L, and 348.5 mg/L Na_2_S_2_O_8_ were 92% (0.0437 min^−1^), 84%, and 81%, respectively [2].

#### 4.3.3 Effect of inlet pressure

Roy *et al.* described that the optimal *P_i_* values using orifice plates with hole diameters of 2, 3, and 4 mm were 10, 1, and 8 bar, respectively [2]. Aseev *et al.* stated that *DEs* of 36 µM CEF by HC/AC/PS (360 µM)/Fe^2+^ (100 µM) increased with increasing *P_i_* values, and the highest *DE* was 81% at 5 bar [14].

#### 4.3.4 Effect of the dosage of Fenton or Fenton-like reagents

Similar to 3.1.3.2, 3.2.3.4, optimal dosages of Fenton reagents also exist. The effect of the dosage of Fenton reagents on pharmaceutical degradation is compiled in Table 9.

**Table 9:** The effect of the dosage of Fenton reagents on pharmaceutical degradation.

Pollutants	Optimal Fe^2+^: H_2_O_2_ ratios	Highest *DEs* (%)	Dosage effects	*Ref.*
Ciprofloxacin	1:3	88	Ratios 1:1 and 1:5 reduced *DEs*	[20]
Oseltamivir phosphate	1:3	68	Ratios 1:1 and 1:5 reduced *DEs*	[4]
Tetracycline	1:10	86	Ratios 1:5 and 1:15 reduced *DEs*	[82]

Note: *DEs*, degradation efficiencies; *Refs*, references.

As shown in Table 9, the optimal Fe^2+^: H_2_O_2_ ratios are in the range of 1:3––1:10, while the appropriate dosage and ratio of Fenton or Fenton-like processes. In addition, Aseev *et al.* described that *DEs* of 36 µM CEF by HC/AC/PS (360 µM)/Fe^2+^ at 50 and 100 μM Fe^2+^, and 5 bar for 2 h were 67% and 78%, respectively, while DEs at higher Fe^2+^ dosages were decreased. They also reported that the *DEs* by HC/AC/PS/Fe^2+^ at the same other conditions were increased by 2.9-fold when increasing the molar PS/CEF ratio from 5:1 to 30:1, leading to the highest *DE* up to 89% in 5 min [14]. Similar to Section 4.3.1, these results further back that HC/AC/Fenton systems are more powerful for pharmaceutical removal.

#### 4.3.5 Effect of initial concentration

*DEs* by HC/AC/PS(360 µM)/Fe^2+^(100 µM) at 5 bar decreased with increasing initial CEF concentration, and the *k* values of CEF removal increased linearly with increasing CEF concentrations from 10 to 50 µM [14].

#### 4.3.6 Effect of co-existing anions and scavenger

${\text{HCO}}_{\text{3}}^{\text{-}}$ can increase the reaction pH, and thus induce the precipitation of Fe^3+^. ${\text{SO}}_{\text{4}}^{\text{2-}}$ can complex with Fe^2+^ and Fe^3+^ through Reactions 90–91, while Cl^−^ can consume ^•^OH to form less powerful ${\text{Cl}}^{∙}$ and HOCl^•^^−^ through Reactions 52 and 56. These anions will inhibit pharmaceutical degradation by HC/Fenton or HC/Fenton-like processes. For instance, Aseev *et al.* found that adding 10 mM ${\text{HCO}}_{\text{3}}^{\text{-}}$, ${\text{SO}}_{\text{4}}^{\text{2-}}$, and Cl^−^ into HC/AC/PS/Fe^2+^ systems inhibited the *DEs* of CEF by ∼ 100%, 15%, and 9%, respectively [14].

MeOH can trap both ${\text{SO}}_{\text{4}}^{∙-}$ and ^•^OH, while t-butanol mainly traps ^•^OH. Adding MeOH and *t*-butanol at an alcohol/PS molar ratio of 500:1in HC/AC/PS/Fe^2+^ systems decreased the *DEs* by 9% and 5%, respectively, leading to the relevant initial *k* values decreased by 1.7 and 1.3 folds. These suggested the crucial roles of ${\text{SO}}_{\text{4}}^{∙-}$ and ^•^OH [14].

## 5 Pharmaceutical removal by hydrodynamic catalysis

### 5.1 Mechanism of hydrodynamic catalysis

Similar to the above-mentioned oxidants and Fenton reagents, catalysts can also intensify the pharmaceutical degradation by HC. Catalysts in HC systems can be dispersible nanoparticles (*e.g.*, metal composites) or coating layers over diodes (*e.g.*, Ag, Cu, Ni, and Fe layers) [20], [21], [94].

### 5.2 Application of hydrodynamic catalysis for pharmaceutical removal

The typical applications of hydrodynamic catalysis processes for pharmaceutical degradation are summarized in Table 10.

**Table 10:** The applications of hydrodynamic catalysis processes for pharmaceutical degradation.

Pollutants	Optimal conditions	*DEs* (%)	*SI* values	*CY* (× 10^-2^ mg/J)	Total costs (USD/m^3^)	*Ref.*
Cephalexin (CFX)	Cu diode-based HC reactor, 20 ppm, 1 bar, 5 min	100	1.2	0.1	0.17	[94]
	1:2,000 M ratio of CFX/H_2_O_2_ with the same other conditions	100	226.5	7.6	0.99	
	pH 9, 1:1,000 M ratio of CFX/H_2_O_2_ with the same other conditions	100	79.6	7.9	0.5	
Cephalexin and ciprofloxacin	Cu diode-based HC reactor, 20 ppm, 1 bar, 3 passes, 1 h	15 and 27	1.2 and 2.1	0.1 and 0.2	−	[22]
	pH 11, 1:1,000 M ratio of antibiotic/H_2_O_2_ with the same other conditions	100 and 47	28.8 and 1.5	∼1.4	0.5	
	1:1,000 M ratio of antibiotic/H_2_O_2_ with the same other conditions	50 and 21	18.7 and 212.5	−	−	
	pH 4, 1:1,000 M ratio of antibiotic/H_2_O_2_ with the same other conditions	63 and 100	2.9 and 11.9	−	−	
Cephalexin and ciprofloxacin	Ni diode-based HC reactor, 3 passes, 20 ppm, 1 bar, 5 min	19 and 37	1.5 and 3.1	0.1 and 0.2	−	[22]
	1:1,000 M ratio of antibiotic/H_2_O_2_ with the same other conditions	100 and 100	188.0 and 153.5	−	−	
	pH 4, 1:1,000 M ratio of antibiotic/H_2_O_2_ with the same other conditions	70 and 100	2.7 and 11.8	−	−	
	pH 11, 1:1,000 M ratio of antibiotic/H_2_O_2_ with the same other conditions	100 and 54	19.6 and 1.5	∼3.7	0.5	
Prazosin (PRH)	Cu diode-based HC reactor, 1 m^3^/h, 20 L, 10 ppm, 0.5 bar	35	1.16	0.5	−	[21]
	Aeration with the same other conditions	∼46	1.43	−	−	
	1:200 M ratio of PRH/H_2_O_2_, 5 min with the same other conditions	100	102.3	13.6	0.0049	
	pH 4 with the same other conditions	100	122.8	13.6	0.0049	

Note: *DEs*, degradation efficiencies; *SI*, synergistic index; *CYs*, cavitation yields; reaction rate constants; *Refs*, references.

As shown in Table 10, the applications of hydrodynamic catalysis for pharmaceutical removal mainly involve studies by Vinay’s group [20], [21], [94], in which high synergistic indexes, remarkably improved *CY* values (up to 1,800%), significantly reduced treatment costs (up to 10-fold), small *P_i_* values (0.5–1.0 bar), and low by-products were reported. Moreover, they stated that Al did not exhibit catalytic activities like Cu and Ni layers, while these layers alone, without pH adjustment and H_2_O_2_ addition, led to limited improvement of the degradation [20], [21], [94]. These results suggest excellent potential of vertex diode-based HC reactors for pharmaceutical degradation, especially with H_2_O_2_ addition and pH change. However, the detailed mechanisms of the Cu- or Ni-induced catalysis in HC systems are still not clear. Although pH adjusting highly reduced the use of H_2_O_2_, the increased use of acidic and basic reagents and the leaching of Cu^2+^ and Ni^2+^ into the treated water matrices are also limitations. A typical example in Fig. 5B shows the CFX degradation by various Cu diode-based HC processes.

### 5.3 Roles of effective factors

#### 5.3.1 Effect of the inlet pressure

Almeida *et al.* reported that *DEs* of PRH decreased when the *P_i_* values exceeded the optimal 0.5 bar, and the Cu-vortex diode led to a *k* value of 7.2 × 10^−3^ min^−1^ at optimal conditions [20].

#### 5.3.2 Effect of aeration

Almeida *et al.* found that aeration into Al and Cu diodes-based HC systems at 0.5 bar improved *DEs* of 10 mg/L PRH from 31% to 39% and from 35% to 46%, respectively, resulting in the relevant *DEs* of TOC being 26% and 35%. Aeration led to SI values of 1.34–1.43. However, the effectiveness of aeration for improved degradation by hydrodynamic catalysis is controversial, depending on the pharmaceuticals and the design of HC reactors [20].

#### 5.3.3 Effect of H_2_O_2_ addition

Almeida *et al.* stated that the lowest molar PRH/H_2_O_2_ ratio among 1:100–1:500 was the optimal H_2_O_2_ dosage for Cu diode-based HC systems, while *DEs* of PRH increased with increasing H_2_O_2_ dosage for Al diode-based HC systems. However, the optimal H_2_O_2_ ratio for PRH mineralization was 1:100, which led to the highest *k* values for PRH degradation of 461 × 10^−3^ and 921 × 10^−3^ min^−1^ for the Al and Cu diodes, respectively [20]. Dixit *et al.* reported that *DEs* of CFX and CIP increased with increasing molar antibiotic/H_2_O_2_ ratios from 1:100 to 1:1,000. At the optimal ratio (*i.e.*, adding 1.9  g/L H_2_O_2_), *DEs* of CEF using Al, Cu, and Ni diodes were 50% in 1 h, 100% in 1 h, and 100% in 5 min, respectively. Compared to the Al diode, using the Ni diode promoted the TOC reduction from 19% to 40% and the *k* values from 11.6 × 10^−3^ to 921 × 10^−3^ min^−1^. Similar results were observed for CIP, with the highest *DEs* of TOC and *k* values of 30% and 1382 × 10^−3^ min^−1^, respectively [22]. In another work, they reported that *DEs* of CFX using Al and Cu diodes increased with increasing H_2_O_2_ dosage from 0.2  g/L to 3.7  g/L. Adding 3.7  g/L H_2_O_2_ led to *DEs* of CEF and TOC using Al diode were 81% (*k* value of 28 × 10^−3^ min^−1^) and 13%, respectively. To achieve 100% of *DEs* using Cu diode for 5 min and 1 h, the required H_2_O_2_ dosages were 3.7 and 1.9 g/L, respectively, and the relevant highest *DEs* of TOC and *k* values were 43% and 31%, as well as 929 × 10^−3^ and 78 × 10^−3^ min^−1^. Fig. S1 displays the HPLC analysis of CFX and by-products in hydrodynamic catalysis systems with and without H_2_O_2_ [94].

In brief, the optimal pharmaceutical/H_2_O_2_ ratios are in the range of 1:100–1:1,000 (or 1.9–3.7 g/L H_2_O_2_), which are related to the nature of coating layers and are case-dependent.

#### 5.3.4 Effect of reaction pH

Almeida *et al.* stated that molecular pharmaceuticals were easy to degrade by HC processes, and low pH favored the removal of PRH due to their high *pK_a_* values of 11.09 and 13.32. At pH 4, *k* values for PRH degradation by Al and Cu diode-based HC were 115.1 × 10^−3^ and 921 × 10^−3^ min^−1^, respectively, and the relevant *DEs* of TOC were 35% and 41% [20]. Dixit *et al.* [21] found that acidic and basic conditions favored the CEF and CIP degradation, respectively. Compared to HC alone, adjusting pH to 11 improved the *DEs* of CEF using Al, Cu, and Ni diodes by 54%, 460%, and 489%, respectively. Similarly, compared to HC alone, adjusting pH to 4 improved the *DEs* of CIP using Al, Cu, and Ni diodes by 185%, 270%, and 170%, respectively [22]. In another work, they also described that high pH favored the CFX degradation, and the highest *k* values were 28.6 × 10^−3^ and 936 × 10^−3^ min^−1^ using Al and Cu diode at pH 11, respectively. Using Cu diode improved the degradation and mineralization of CFX by 22% and 115%, respectively. The relevant HPLC analysis and LC-MS spectra for CFX degradation are demonstrated in Fig. S1 and S2, respectively [94]. These results once again highlight the imperative roles of pH change in diode-based HC systems.

## 6 Pharmaceutical removal by HC/Ozonation

### 6.1 Mechanism of HC/Ozonation

O_3_ exhibits both electrophilic and nucleophilic properties. O_3_ can selectively break down double bonds in organics, thus some pharmaceuticals are resistant to O_3_. The selective oxidation of O_3_ could be an advantage or a drawback, depending on the properties of target pharmaceuticals. Introducing O_3_ into HC systems can enhance ROS production via Reactions 33–48, inducing non-selective oxidation. In turn, HC can improve the dissolution of O_3_ via Reactions 30–32 due to the formation of O_3_ ultrafine bubbles in large numbers and the intensified mass transfer, which can further improve radical generation [3], [23], [24]. For instance, Huang *et al.* reported that HC/O_3_ improved the ^•^OH production by 60-fold compared to O_3_ alone [23]. Hence, HC/O_3_ favors the decomposition of O_3_-persistence pharmaceuticals in water matrices. Mass transfer of O_3_ gases and various ROS is especially critical in HC/O_3_ processes. However, the reaction conditions of combined HC/O_3_ systems should be carefully optimized, as introducing O_3_ may influence the formation, nature, and dynamics of cavitation bubbles (*e.g.*, residence time, the composition and proportion of gases, size and amounts of bubbles) and the HC effects, and the HC conditions may also affect the O_3_ utilization and ozonation performance in turn. In addition, introducing O_3_ into HC systems may cause the production of undesired intermediates such as bromate and trihalomethanes [97].

### 6.2 Application of HC/Ozonation for pharmaceutical removal

The typical applications of HC/O_3_ and HC/photocatalysis for pharmaceutical degradation are summarized in Table 11.

**Table 11:** The applications of HC/O_3_ and HC/Photocatalysis for pharmaceutical degradation.

Pollutants	Optimal conditions	*DEs (%)*	*SI* values	Other results	*Ref.*
Chlortetracycline (CTC)	Orifice-based reactor, 6 L, 10 ppm CTC, 0.5 g/L CuFe_2_O_4_/Fe_2_O_3_, 20℃, 4 bar, 1 h	82	−	Heat generation and thermal efficiency in 0.5 h were 1481.3 KJ and 54.9%, respectively	[81]
Ciprofloxacin	Orifice-based reactor, 1 g/L, sludge/TiO_2_, 4 bar, 1 h	74	∼1.27	−	[113]
Doxycycline (DOX)	Venturi-based reactor, 5 L, 30 ppm DOX, 0.5 g ZnIn_2_S_4_/NiFe_2_O_4_/biochar, pH 5, 4 bar, 1 h	97	2.70	−	[92]
Oseltamivir phosphate (OP)	Venturi-based HC reactor, 10 ppm OP, 3 g/h O_3_, 25°C, 0.5 h	95	3.5	*CY* and treatment cost were 2.7 × 10^−4^ mg/J and 2.2 × 10^−4^ USD/(L.mg), respectively	[4]
Oxytetracycline (OTC)	Venturi-based reactor, 4 μM OTC, gas–liquid ratio of 0.06, 23.6°C, 3 s	95	−	−	[23]
Pefloxacin (PEF)	10 ppm PEF, pH 5.3, 3 bar, 0.675 g/h, 0.33 h	92	2.19	*−*	[24]

Note: *DEs*, degradation efficiencies; *SI*, synergistic index; *Refs*, references.

As shown, introducing O_3_ into HC systems improved the degradation of pharmaceuticals. Katiyar *et al.* reported that the treatment cost of HC/O_3_ was decreased by 218%, 203%, and 165% compared to HC alone, HC/Fenton, and HC/H_2_O_2_, respectively [4]. However, Liu *et al.* reported that although HC/H_2_O_2_/O_3_ can improve the CFX degradation, it increased the treatment cost by 45-fold compared to HC alone [31]. Mukherjee *et al.* also stated that introducing O_3_ into HC systems increased the CY, energy consumption, and treatment cost for CIP degradation by 110%, 89%, and 91%, respectively [19]. The energy consumption is in the range of 0.12–61.00 kWh/m^3^ (or kWh/mg) [2], [24], [97]. A typical example in Fig. 5C shows the OTC degradation by HC/O_3_.

### 6.3 Roles of effective factors

#### 6.3.1 Degradation by various processes

**Fig. 6:**
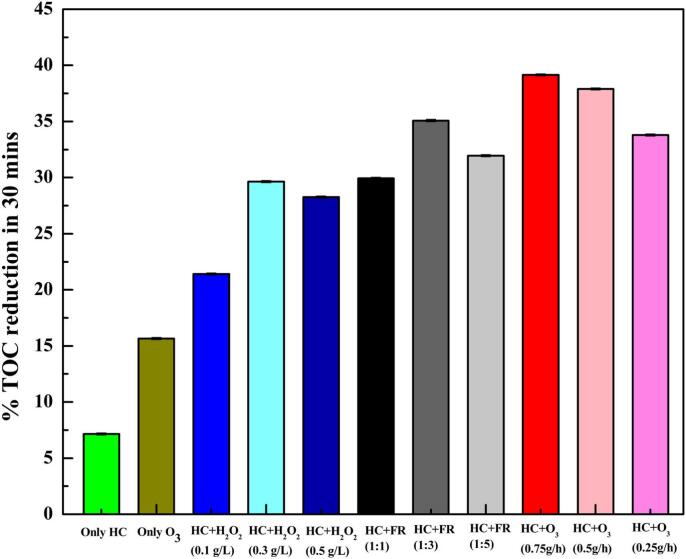
TOC reduction during CIP degradation by various processes for 0.5 h. Reprinted from ref. [19]. Copyright (2021), with permission from Elsevier. Note: FR refers to the Fenton reaction.

High *SI* values in Table 11 confirm the great pharmaceutical decomposition ability of HC/O_3_ compared to individual HC and O_3_ processes. Mukherjee *et al.* stated that HC/O_3_ led to higher mineralization of CIP than HC/H_2_O_2_ and HC/Fenton processes, even in real wastewater at a pilot scale (400 L) (Fig. 6) [19]. Moreover, Devia-Orjuela *et al.* incorporate UV irradiation in HC/O_3_ systems, resulting in higher DEs than the relevant individual processes [97].

#### 6.3.2 Degradation of different pharmaceuticals

Pharmaceuticals’ properties (Table S1), such as hydrophobicity and solubility, can influence their interactions with O_3_ and ROS, resulting in different degradation performances [3], [31].

Kumar *et al.* investigated the degradation of various pharmaceuticals in real effluents by HC/O_3_ for 30 s at a pilot-scale in flow mode using an orifice-nozzle based HC reactor at 30 bar and a *C_v_* of 0.03. At O_3_ dosage of 1 and 7 mg/L, *k* values for 1H-Benzotriazole, venlafaxine, and metoprolol were 0.3, 0.4, and 0.5 min^−1^, respectively. At O_3_ dosage of 7 mg/L, *DEs* can be ordered as metoprolol > venlafaxine > 1H-benzotriazole > MET [3]. Huang *et al.* reported that 95–118 μg/L OTC, TC, CTC, SDZ, SMZ, SMX, CHL, and FLO in simulated aquaculture seawater were degraded to an undetectable level by HC/O_3_ in 20 s [25]. Our previous work found better degradation performance of hydrophobic marbofloxacin and OTC by HC/O_3_ for 40 min at an initial antibiotic concentration of 5.52 μM than hydrophilic ceftiofur and sulfamonomethoxine in 400 mL milk [31].

#### 6.3.3 Effect of operating time

Usually, longer operating time means higher retention time of pharmaceutical-containing water matrices in HC/O_3_ systems, followed by increased exposure of pharmaceutical molecules to O_3_ and ROS, thus leading to better degradation [3].

Kumar *et al.* stated that an additional pass increased the *DEs* of 1H-Benzotria by 75%, Metoprolol by 38%, and Venlafaxine by 36% using HC/O_3_ (7 mg/L) [3]. However, some authors reported that prolonged operating time of HC/O_3_ did not lead to better degradation of pharmaceuticals but resulted in improved mineralization, and they attributed this to the competitive reaction between pharmaceutical molecules and intermediates with oxidative species in HC/O_3_ systems [23], [24].

#### 6.3.4 Effect of O_3_ dosage

Increasing O_3_ dosage will lead to more ^•^OH generation, favoring the pharmaceutical degradation. However, excessive O_3_ input may trigger more generation of O_3_ bubbles in HC systems, which will weaken the cavitation intensity, leading to decreased degradation [4].

Katiyar *et al.* reported that *DEs* of OP by HC/O_3_ increased with increasing O_3_ dosage from 1 to 3 g/h, but decreased at higher O_3_ dosage. The highest *k* value at 3 g/h was 0.104 min^−1^ [4]. Mukherjee *et al.* reported that higher O_3_ dosage led to higher *DEs* of CIP in HC/O_3_ processes. Increasing dosage from 0.25 to 0.75  g/h resulted in an increase in *DE* from 78.2% to 91.4%, and an increase in *k* values from 5.6 × 10^−^^2^ to 9.3 × 10^−^^2^ min^−1^ [19]. Wang *et al.* [23] stated that increasing O_3_ dosage from 0.059 to 0.675 g/h increased *DEs* of PEF by HC/O_3_ [24]. Huang *et al.* reported that *DEs* of OTC decreased linearly with increasing the gas–liquid ratio from 0.012 to 0.060 in HC/O_3_ systems, while *DEs* were significantly reduced when the ratio was over 0.06. They stated that the mass transfer of gases O_3_ to liquids was crucial to ensure high OTC degradation rates. Fig. S3 demonstrates the LC-MS spectra for OTC degradation by-products for HC/O_3_ systems with different gas–liquid ratios [23]. In another work, they reported that *DEs* of FLO by HC/O_3_ for 0.6 s were ∼ 70%, and *k* values increased from 7 × 10^-2^ to 17 × 10^-2^ min^−1^ when the gas–liquid ratio increased from 0.006 to 0.024 [25]. Kumar *et al.* reported that adding 1 and 7 mg/L O_3_ in HC systems led to an increase in *DEs* from 33% to 67% for 1H-Benzotriazole, 92% to 131% for metoprolol, and 214% to 300% for venlafaxine, while *DEs* of MET increased from 0 to ∼ 9%. The author attributed the improved degradation compared to HC alone to the increased production of bubbles, the influence of O_3_ on the cavitation behavior, the enhanced mass transfer and reaction dynamics, as well as the promoted ROS formation [3].

#### 6.3.5 Effect of pH and initial pharmaceutical concentrations

It has been reported that O_3_ directly interacts with pharmaceuticals at low pH, while it will indirectly oxide pharmaceuticals at high pH through ^•^OH. However, the optimal pH is case-dependent owing to the change of molecular forms with pH [24], [97].

Devia-Orjuela *et al.* reported that high pH (>6) led to higher *DEs* of CBZ by HC/O_3_, due to the increased generation of ^•^OH and the improved interactions of OH and ionized CBZ. They also found that *DEs* > 14% when initial CBZ concentrations exceeded 6 g/m^3^ [97]. However, Huang *et al.* also described that acidic and natural conditions favored the removal of 4 μM OTC by HC/O_3_. They stated that ^•^OH and ${\text{O}}_{2}^{∙-}$ (which is ineffective for OTC removal) were dominated under acidic and basic conditions in the current case, respectively [23]. In another work, they found that the reaction pH increased after treating various antibiotics with HC/O_3_, and they attributed this to the oxidation of some acidic organics [25].

## 7 Pharmaceutical removal by HC/Photocatalysis

### 7.1 Mechanism of HC/Photocatalysis

**Fig. 7:**
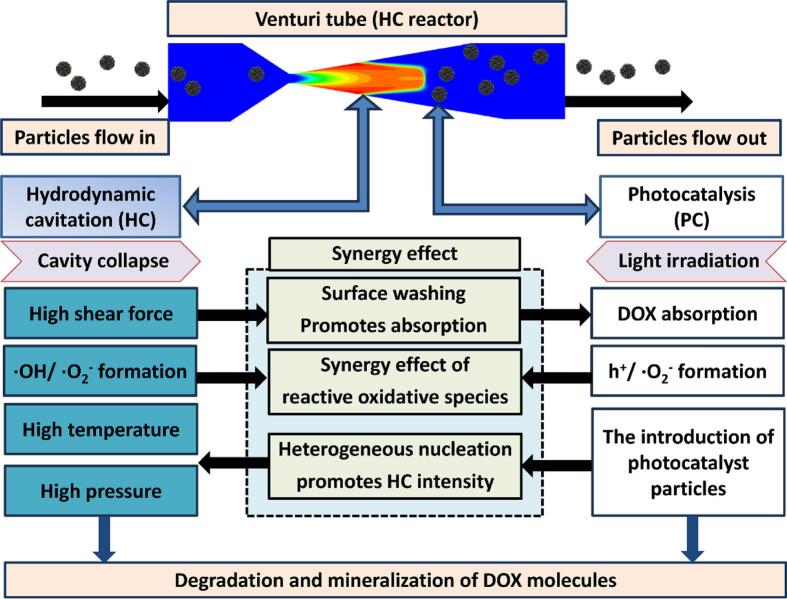
Mechanism of HC/Photocatalysis. Reprinted from ref. [92]. Copyright (2025), with permission from Elsevier.

Photocatalysis works based on the actions of ${\text{O}}_{2}^{∙-}$, ^•^OH, and photo-induced holes *(h*^*+*^*)* and *e^−^* generated by activating semiconductor catalysts under photo irradiation through Reactions 92–95 (see Supplementary materials). This process can be limited by the photon transmittance of water matrices, the structure and physicochemical properties of photocatalysis, the inactivation, regeneration, and recovery of photocatalysis, the characterization of light sources, poor mass transfer, and the expensive running and maintenance [16], [113], [114]. HC can intensify the photocatalysis by increasing the ROS formation without adding extra chemical reagents, mitigate photocatalyst aggregation through the circular flow-induced fluid disturbance, and maintain their catalytic activity via continuously refreshing their surface to remove the passivation layer and adsorbed intermediates on the active sites by high-speed micro-jets and shock waves. On the other hand, adding photocatalysts can also improve the nucleation of cavitation bubbles thanks to their carried gases, and decrease cavitation threshold, allowing intensified cavitation effects. However, photocatalysts at high dosage in HC systems will result in the decrease of total surface area due to the solid aggregation and reduce the photon depth penetration (Fig. 7). Overall, incorporating HC in photocatalysis is expected to synergistically enhance the degradation performance with reduced treatment costs and improved energy efficiency. The main limitations of HC/Photocatalysis are the possible secondary pollution and the recovery of the used catalysts [92], [100], [115].

### 7.2 Application of HC/Photocatalysis for pharmaceutical removal

The typical applications of HC/Photocatalysis for pharmaceutical degradation are summarized in Table 11. As shown, combined HC/Photocatalysis led to high DEs of various pharmaceuticals with large *SI* values. Wu *et al.* found that *DEs* of DOX by HC/Photocatalysis at the second and third passes were reduced by 4.2% and 7.3%, respectively. They attributed this decrease to the blockage of the catalyst’s pores and the changed surface properties [92].

### 7.3 Roles of effective factors

#### 7.3.1 Degradation by various processes

Similarly, high *SI* values in Table 11 indicate the outstanding pharmaceutical degradation ability by HC/Photocatalysis processes compared to the relevant individual processes. Furthermore, Liu *et al.* found that adding 1 mM H_2_O_2_ in HC/UV systems increased the *DEs* of TC by 2-fold (73%) with a *SI* value of 4.9, while adding 1 mM H_2_O_2_ in UV alone systems increased *DEs* by 93% with a *SI* of 3.0 [100]. Therefore, there is no doubt that H_2_O_2_ is of great importance in the improvement of the pharmaceutical degradation performances in HC alone, hydrodynamic catalysis, HC/Fenton, HC/O_3_, and HC/Photocatalysis systems. Nevertheless, it is complex and difficult to point out the most effective strategies among these systems. The optimal approaches not only depend on the *DEs* but also on the cavitation yields, treatment costs, and energy consumption. Tailoring the exclusive systems for each case is necessary and crucial for addressing the pharmaceutical-induced water pollution spreading.

#### 7.3.2 Effect of reaction pH

Wu *et al.* [92] stated that acidic conditions favor DOX degradation by HC/Photocatalysis for 1 h. *DEs* were 99%, 98%, 81%, 61%, and 55% at pH of 3, 5, 7, 9, and 11, respectively. pH changed the formation of ^•^OH, affected the existing form of DOX (p*K*_a_ are 3.5, 7.7, and 9.5), and influenced the DOX adsorption by catalysts [92]. Similar results were reported by Liu *et al.*, and lower pH 3 led to better TC degradation than higher pH 10 by HC/UV/H_2_O_2_ (1 mM) at 30 mg/L, 3.5 bar, 25℃, and 8 W × 4 lamp for 1 h [100].

#### 7.3.3 Effect of co-existing ions and scavengers

Wu *et al.* [92] stated that 10 mg/L Ca^2+^ and Mg^2+^ can complex with DOX molecules, and the latter has a higher inhibition efficiency of DOX degradation, especially at high ion concentrations of 15–20 mg/L. Similar results were observed with adding 10–20 mg/L anions, and the inhibition efficiencies ordered as humic acid anions < Cl^−^ < ${\text{NO}}_{\text{3}}^{\text{-}}$ < ${\text{CO}}_{\text{3}}^{2\text{-}}$. They prove that ${\text{O}}_{2}^{∙-}$, ^•^OH, and h^+^ were the main species for DOX degradation in HC/Photocatalysis systems using dimethyl sulfoxide, benzoquinone, and ethylenediaminetetraacetic acid disodium, respectively. ^•^OH accelerated the breakdown of carbon rings, and ${\text{O}}_{2}^{∙-}$ and h^+^ disrupted various chemical groups [92]. Nevertheless, Noori *et al.* reported that adding Cl^−^ in HC/Photocatalysis systems improved the atenolol degradation [89].

## 8 Environmental impacts and sustainability

Compared to other AOPs, the distinctive environmental impacts induced by various HC processes for pharmaceutical removal are improved energy efficiency, chemical usage, and secondary waste generation, especially for the vortex diode-based HC processes. It is well known that HC processes have higher energy effectiveness and industrialization potential compared to sonochemical processes. HC can produce oxidants like H_2_O_2_ and ^•^OH itself, meaning it can be a chemical-free process, significantly reducing the environmental burden associated with chemical production, transport, storage, and residual in the effluent. The excellent degradation and mineralization of hazardous pharmaceuticals by hybrid HC processes avoid the harmful effects of them on the environment and the possible risk of secondary pollution. The synergistic effects in HC/H_2_O_2_, HC/PS (or PMS), HC/PCB, HC/Fenton, HC/O_3_, HC/Photocatalysis, and HC/Plasma processes further promoted the positive environmental impacts owing to the accelerated treatment, short running period, and less use of additives. However, the cost and energy consumption, the use of H_2_O_2_, Fenton reagents, catalysts, UV emitter, O_3_ generator, and plasma discharger, *etc.*, may be increased in hybrid HC processes, requiring careful optimization of these systems. The produced ferric hydroxide sludge and the release of Fe^2+^ and Fe^3+^ ions in the treated water during the combined HC/Fenton processes may induce negative environmental impacts. Additionally, the low mineralization in individual HC systems may produce certain intermediates with higher toxicity than their parent pharmaceutical compounds [5], [16], [44].

Life Cycle Assessment (LCA) can evaluate the sustainability of various HC processes for pharmaceutical removal. Generally, the environmental benefit gained from significantly increased pollutant removal and mineralization (avoiding future ecosystem damage) must outweigh the environmental cost of the increased energy or chemical input of the hybrid system. In many reported cases, hybrid HC processes, when properly optimized, are found to be a sustainable and cost-effective approach for the degradation of persistent organic pollutants like pharmaceuticals, offering a better compromise between treatment efficiency and environmental load compared to non-HC AOPs.

In detail, from the perspective of LCA, the environmental profile in an individual HC system is almost entirely sensitive to the electricity mix. If the grid is coal-heavy, the Global Warming Potential is dominated by the specific energy consumption required to reach the target degradation, often exceeding 50–100 kWh/m^3^. However, in the case of a hybrid HC process, such as HC/Fenton, it typically reduces the mechanical treatment time by 50%–70%, lowering direct electricity use. The “sustainability trade-off” arises when accounting for the chemical production burdens: the synthesis of H_2_O_2_ is energy-intensive (anthraquinone process), often contributing up to 40% of the hybrid's total Global Warming Potential. Furthermore, the sensitivity to the electricity mix is more pronounced in the HC-only setup; in regions with green grids (*e.g.*, Norway or Brazil), HC-only becomes significantly more sustainable than hybrids because it avoids the “frozen” carbon costs of chemical reagents and the secondary pollution of iron sludge [92]. While hybrids offer faster kinetics, their lifecycle sustainability is frequently inferior unless the catalyst can be recovered or the synergy reduces energy demand by an order of magnitude [116], [117], [118].

## 9 Challenges and future perspectives

Individual HC processes generally suffer from poor degradation and mineralization efficiencies, and strong sensitivity to process parameters and reactor dependence. Although additives like H_2_O_2_, PS, PMS, and PCB favor the process intensification and the optimization of energy efficiency, they often increase operational costs. Hence, achieving high synergistic effects in HC/Oxidant processes is critical for effective pharmaceutical removal. Key challenges for hybrid HC processes include the development of advanced reactors (*e.g.*, vortex diode- and rotor-based reactors) with large cavitation zones and high energy efficiency, and *CY* values to enable rapid degradations and toxicity reduction. Moreover, the scalability, sustainability, and potential negative environmental impacts must be carefully evaluated.

Promising future strategy involves using hybrid HC processes as pre-treatment of pharmaceuticals to reduce the toxicity and enhance biodegradability, followed by conventional and cost-effective biological treatment for large volume pharmaceutical-contaminated wastewater. The use of waste streams, such as the byproducts from other treatment processes, as a “carrier” liquid for HC systems can further promote circular economy principles. In addition, computational fluid dynamics offers valuable insight into pressure distribution, bubble dynamics, and chemical reaction mechanisms, facilitating rational reactor design, optimization, and reactor scale-up. Bhandari *et al.* recommended the per-pass degradation model to evaluate the degradation, which can effectively capture both the physical and chemical aspects of HC processes [5], [16]. Last but not least, conducting more pilot-scale and industrial-scale studies to confirm the energy efficiency, robustness, and long-term cost-effectiveness of HC and hybrid HC systems in real-world wastewater treatment plants. Overall, HC, especially in hybrid configurations, is a promising and scalable green technology for tackling recalcitrant pharmaceutical pollutants. The future lies in developing optimized, energy-efficient reactors and intelligently integrating HC with other processes to achieve complete, cost-effective mineralization and toxicity reduction.

## 10 Conclusions

Individual HC processes are often insufficient for effective pharmaceutical degradation in water matrices. Optimal inlet pressures for Venturi-, orifice-, SEOC-, and vortex diode-based reactors are 2.5–4.0 bar, 5 bar, 5 bar, and 1.0–1.5 bar, respectively, maximizing cavitation intensity and degradation performance. In most cases, pH 2–5 are conducive to the pharmaceutical removal.

In contrast, integrating HC with oxidants, Fenton reagents, catalysts, ozonation, photocatalysis, and plasma can significantly enhance the *RE*. Appropriate reactor design and optimization of HC reactor’s operating conditions are essential to achieve strong cavitation effects, efficient oxidation activation, and effective interactions between HC, additives, and catalysts. whereas high dosages of oxidative additives do not always improve removal. Reaction pH strongly influences pharmaceutical speciation, ROS generation, and oxidative strength, and the spatial distribution of contaminants relative to cavitation bubbles, particularly in diode-based HC systems. Moderately higher initial pharmaceutical concentration can simultaneously improve both *CY* and *DEs* values, while shortening treatment duration. Anions are generally recommended to be removed from water matrices prior to treatment to avoid the antagonistic effect. But ${\text{CO}}_{\text{3}}^{\text{2-}}$ and ${\text{HCO}}_{\text{3}}^{\text{-}}$ ions may, under certain conditions, exert positive effects. O_3_ injection could influence bubble formation and cavitation dynamics, potentially impairing degradation performance. Reactive species such as ^•^OH, H_2_O_2_, ${\text{SO}}_{\text{4}}^{∙-}$, O_3_, ${\text{O}}_{2}^{∙-}$, and h^+^ play key roles in pharmaceutical degradation across various HC processes. The optimal approaches are identified by the degradation performance, the cavitation yields, treatment costs, and energy consumption. Tailoring the exclusive systems for each case is necessary and crucial for addressing the pharmaceutical-induced water pollution spreading.

Overall, mass transfer, cavitation intensity, the generation of ROS, and the interactions of ROS with pharmaceutical molecules are of great importance, regardless of the HC systems.

## Author contributions

The manuscript was written through the contributions of all authors. All authors have given approval to the final version of the manuscript.

## CRediT authorship contribution statement

**Pengyun Liu:** Writing – original draft, Investigation, Data curation. **Emanuela Calcio Gaudino:** Investigation, Formal analysis, Conceptualization. **Judy Lee:** Writing – original draft, Supervision, Conceptualization. **Giancarlo Cravotto:** Writing – review & editing, Supervision, Methodology, Conceptualization.

## Declaration of competing interest

The authors declare that they have no known competing financial interests or personal relationships that could have appeared to influence the work reported in this paper.
