# Supplementary material for: A review of pharmaceutical removal from wastewater via hydrodynamic cavitation
Source: Ultrason Sonochem. 2026 Apr 1;129:107843. doi: 10.1016/j.ultsonch.2026.107843 (PMC13091153; doi:10.1016/j.ultsonch.2026.107843)
Supplement: Supplementary Data 1 [file mmc1.docx]

**A review of pharmaceutical removal from wastewater via hydrodynamic cavitation**

Pengyun Liu^a^, Emanuela Calcio Gaudino^a^, Judy Lee^b,^*, Giancarlo Cravotto^a,^*

^a^ Department of Drug Science and Technology, University of Turin, via P. Giuria 9, 10125 Turin, Italy.

^b^ School of Chemistry and Chemical and Process Engineering, University of Surrey, Guildford, GU2 7XH, UK.

*Correspondence: [giancarlo.cravotto@unito.it](mailto:giancarlo.cravotto@unito.it); [j.y.lee@surrey.ac.uk](mailto:j.y.lee@surrey.ac.uk); Tel: +39.011.670.7183; Fax: +39.011.670.7162.

**Supporting Reactions**

H_2_O $\underset{\to}{\text{ HC }}$^•^OH + ^•^H (1)

H_2_O $\underset{\to}{\text{ HC }}$H^+^ + ^•^OH + e^-^ (2)

^•^OH + ^•^OH $\leftrightarrow$ H_2_O_2_ (3)

H_2_O_2_ + ^•^OH → ${\text{H}\text{O}}_{\text{2}}^{\bullet}$ + H_2_O (4)

${\text{H}\text{O}}_{\text{2}}^{\bullet}$ + ${\text{H}\text{O}}_{\text{2}}^{\bullet}$ → H_2_O_2_+ O_2_ (5)

^•^OH +${\text{H}\text{O}}_{\text{2}}^{\bullet}$ → H_2_O_2_ (6)

^•^OH + ${\text{H}\text{O}}_{\text{2}}^{\bullet}$ → H_2_O + O_2_ (7)

^•^OH + O_2_$\underset{\to}{\text{HC }}$ ${\text{H}\text{O}}_{\text{2}}^{\bullet}$ + ^•^O (8)

H_2_O_2_ + ^•^H → H_2_O + ^•^OH (9)

H_2_O_2_ + ^•^OH → H_2_O + ${\text{H}\text{O}}_{\text{2}}^{\bullet}$ (10)

H_2_O_2_ + ^•^OH → H_2_O + $\text{O}_{\text{2}}^{\bullet\text{-}}$ + H^+^ (11)

H_2_O_2_ + $\text{O}_{\text{2}}^{\bullet\text{-}}$ → ^•^OH + OH^-^ + O_2_ (12)

H_2_O_2_ + ${\text{H}\text{O}}_{\text{2}}^{\bullet}$→ ^•^OH + H_2_O + O_2_ (13)

H_2_O_2_ + *e*^-^ → ^•^OH + OH^-^ (14)

$\text{O}_{\text{2}}^{\bullet\text{-}}$ + H^+^ $\leftrightarrow$ ${\text{H}\text{O}}_{\text{2}}^{\bullet-}$, p*K*_a_ = 4.8 (15)

$\text{O}_{\text{2}}^{\bullet\text{-}}$+ ^•^OH → OH^-^ + O_2_ (16)

$\text{O}_{\text{2}}^{\bullet\text{-}}$ + ^•^OH → ^1^O_2_ + OH^-^ (17)

$\text{O}_{\text{2}}^{\bullet\text{-}}$ + ^•^OH + 2 *e^-^* → OH^-^ + ^1^O_2_ (18)

$\text{O}_{\text{2}}^{\bullet\text{-}}$ + ${\text{H}\text{O}}_{\text{2}}^{\bullet\text{-}}$ + H^+^ → ^1^O_2_ + H_2_O_2_ (19)

O_2_ → 2O^•^ (20)

O_2_ + ^•^H → ^•^OH + O^•^ (21)

^•^O + H_2_O$\underset{\to}{\text{HC }}$ 2^•^OH (22)

S_2_$\text{O}_{\text{8}}^{\text{2-}}$ $\underset{\to}{\text{OH}^{\text{-}}/\text{thermal/cavitation}}$ 2$\text{SO}_{\text{4}}^{\bullet\text{-}}$ (23)

S_2_$\text{O}_{\text{8}}^{\text{2-}}$ + $\text{SO}_{\text{4}}^{\bullet\text{-}}$ → $\text{SO}_{\text{4}}^{\text{2-}}$ + S_2_$\text{O}_{\text{8}}^{\bullet\text{-}}$ (24)

S_2_$\text{O}_{\text{8}}^{\text{2-}}$ + *e^-^* → $\text{SO}_{\text{4}}^{\text{2-}}$ + $\text{SO}_{\text{4}}^{\bullet\text{-}}$ (25)

2$\text{SO}_{\text{4}}^{\bullet\text{-}}$ $\underset{\to}{\text{H}^{\text{-}}/\text{thermal/cavitation}}$ S_2_$\text{O}_{\text{8}}^{\text{2-}}$ (26)

$\text{SO}_{\text{4}}^{\bullet\text{-}}$ + OH^−^ $\leftrightarrow$ $\text{SO}_{\text{4}}^{\text{2-}}$ + ^•^OH (27)

$\text{SO}_{\text{4}}^{\bullet\text{-}}$ + H_2_O → $\text{SO}_{\text{4}}^{\text{2-}}$ + ^•^OH + H+ (28)

$\text{SO}_{\text{4}}^{\bullet\text{-}}$ + H_2_O → ${\text{H}\text{SO}}_{\text{4}}^{\text{-}}$ + ^•^OH (29)

O_3_ (gas) $\underset{\to}{\text{ }\text{HC}\text{ }}$O_3_ (liquid) (30)

O_3_ (liquid) + H_2_O$\underset{\to}{\text{HC }}$ ^•^OH + O_2_ (31)

O_3_ (liquid)$\underset{\to}{\text{HC }}$ O_2_ + ^•^O (32)

O_3_ + H_2_O_2_ → ^•^OH + ${\text{H}\text{O}}_{\text{2}}^{\bullet}$ + 0.5 O_2_ (33)

O_3_ + $\text{O}_{\text{2}}^{\bullet\text{-}}$ → $\text{O}_{\text{3}}^{\bullet\text{-}}$ + O_2_ (34)

O_3_ + 2 H^+^ + 2*e^-^* → O_2_ + H_2_O (36)

O_3_ + ^•^OH + 2*e^-^*→ ${\text{H}\text{O}}_{\text{2}}^{\bullet}$ + $\text{O}_{\text{2}}^{\bullet\text{-}}$ (37)

O_3_ + OH^-^ →${\text{H}\text{O}}_{\text{2}}^{-}$ + O_2_ (38)

O_3_ + ${\text{H}\text{O}}_{\text{2}}^{\bullet}$ → ^•^OH + 2O_2_ (39)

O_3_ + H_2_O_2_ → O_2_ + ^•^OH + ${\text{H}\text{O}}_{\text{2}}^{\bullet}$ (40)

O_3_ + H_2_O_2_ → 2^•^OH + 3O_2_ (41)

H_2_O_2_ $\leftrightarrow$ ${\text{H}\text{O}}_{\text{2}}^{-}$ + H^+^ (42)

$\text{HO}_{\text{2}}^{\text{-}}$ + O_3_ → $\text{HO}_{\text{5}}^{\text{-}}$ (43)

$\text{HO}_{\text{2}}^{\text{-}}$ + ^•^OH → ${\text{H}\text{O}}_{\text{2}}^{\bullet-}$ + H_2_O (44)

$\text{HO}_{\text{5}}^{\text{-}}$ → ${\text{H}\text{O}}_{\text{2}}^{\bullet}$ + $\text{O}_{3}^{-}$ (45)

$\text{O}_{\text{3}}^{\text{-}}$ + H_2_O → O_2_ + ^•^OH + OH^-^ (46)

$\text{O}_{\text{3}}^{\bullet\text{-}}$ $\leftrightarrow$ O^•-^+ O_2_ (47)

O^•-^+ H_2_O $\leftrightarrow$^•^OH + OH^-^ (48)

$\text{CO}_{\text{3}}^{\text{2-}}$ + ^•^OH→ OH^-^ + $\text{CO}_{\text{3}}^{\bullet\text{-}}$ (49)

$\text{CO}_{\text{3}}^{\text{2-}}$ + ^•^OH → H_2_O_2_+ $\text{CO}_{\text{3}}^{\bullet\text{-}}$ (50)

$\text{CO}_{\text{3}}^{\bullet\text{-}}$ + $\text{O}_{\text{3}}^{\bullet\text{-}}\text{ }$→ $\text{CO}_{\text{3}}^{\text{2-}}$ + O_3_ (51)

Cl^-^ + ^•^OH $\leftrightarrow$ HOCl^•-^ (52)

Cl^-^ + O_3_ → O_2_ + OCl^-^ (53)

OCl^-^ + O_3_ → 2O_2_ + Cl^-^ (54)

OCl^-^ $\leftrightarrow$ HOCl (p*K*_a_ 7.5) (55)

HOCl^•-^ + H^+^ $\leftrightarrow$ Cl^•^ + H_2_O (56)

HOCl^•-^ + Cl^-^$\leftrightarrow$ $\text{Cl}_{\text{2}}^{\bullet\text{-}}$ + OH^-^ (57)

Cl^•^ + OH^-^ → ClOH^•-^ (58)

Cl^•^ + Cl^-^ → $\text{Cl}_{\text{2}}^{\bullet\text{-}}$ (59)

Cl^•^ + OCl^-^ → H^+^ + Cl^-^ + ClO^-^ (60)

$\text{SO}_{\text{4}}^{\text{2-}}$ + OH^•^→ $\text{SO}_{\text{4}}^{\bullet-}$ + OH^-^ (61)

^•^OH + $\text{PO}_{\text{4}}^{\text{3-}}$ → OH^-^ + $\text{PO}_{\text{4}}^{2\bullet\text{-}}$ (62)

^•^OH + $\text{HPO}_{\text{4}}^{\text{2-}}$ → H_2_O + $\text{PO}_{\text{4}}^{2\bullet\text{-}}$ (63)

^•^OH +$\text{NO}_{\text{3}}^{\text{-}}$ → $\text{NO}_{\text{3}}^{\bullet}\text{ }$+ H_2_O (64)

^•^OH + H_2_O_2_ → H_2_O + $\text{O}_{\text{2}}^{\bullet\text{-}}$ + H (65)

$\text{SO}_{\text{4}}^{\text{2-}}\text{ }$+ ^•^OH → $\text{SO}_{\text{4}}^{\bullet-}$ + OH^-^ (66)

${\text{S}\text{O}}_{\text{4}}^{\bullet\text{-}}$ + H_2_O → ^•^OH +${\text{HS}\text{O}}_{\text{4}}^{\text{-}}$ (67)

$\text{NO}_{\text{3}}^{\text{-}}$ + ^•^OH→ OH^-^ + $\text{NO}_{\text{3}}^{\bullet}$ (68)

$\text{NO}_{\text{3}}^{\text{-}}$ + H_2_O → ^•^OH +OH^-^ + $\text{NO}_{\text{3}}^{\bullet}$ (69)

I^-^ + ^•^OH→ I^•^ + OH^-^ (70)

$\text{HCO}_{\text{3}}^{\text{-}}$ + $\text{SO}_{\text{4}}^{\text{2-}}$ → H_2_O + $\text{CO}_{\text{3}}^{\bullet-}$ (71)

2$\text{CO}_{\text{3}}^{\bullet\text{-}}$ + H_2_O → CO_2_ + OH^-^ + ${\text{H}\text{O}}_{\text{2}}^{\text{-}}$ (72)

$\text{SO}_{\text{4}}^{\text{•-}}+\text{Cl}^{\text{-}}\text{⇌}\text{Cl}^{\text{•}}+\text{SO}_{\text{4}}^{\text{2-}}$ (73)

$\text{SO}_{\text{4}}^{\text{•-}}+\text{N}\text{O}_{\text{3}}^{\text{-}}\to\text{SO}_{\text{4}}^{\text{2-}}+\text{NO}_{\text{3}}^{\bullet}$ (74)

$\text{SO}_{\text{4}}^{\text{•-}}$ + $\text{HC}\text{O}_{\text{3}}^{\text{-}}\text{→}\text{CO}_{\text{3}}^{\bullet-}+\text{H}\text{2}\text{O}$ (75)

$\text{HSO}_{\text{5}}^{\text{-}} \underset{\to}{\text{ }\text{HC }}$ $\text{SO}_{\text{4}}^{\text{•-}}$ + ^•^OH (76)

2$\text{SO}_{\text{4}}^{\text{•-}}$ + 2^•^OH → 2$\text{HSO}_{\text{4}}^{\text{-}}$ + O_2_ (77)

^•^OH + OH^−^ → H_2_O + O^•−^ (78)

${\text{H}\text{CO}}_{\text{3}}^{\text{-}}$ + H_2_O_2_ → ${\text{H}\text{CO}}_{4}^{\text{-}}$ + H_2_O (79)

${\text{H}\text{CO}}_{4}^{\text{-}}$ → $\text{CO}_{\text{3}}^{\bullet\text{-}}$ + H^+^ (80)

$\text{CO}_{\text{3}}^{\bullet\text{-}}$ + H_2_O → ${\text{H}\text{CO}}_{\text{3}}^{\text{-}}$ + ${\text{H}\text{O}}_{\text{2}}^{\bullet}$ (81)

${\text{H}\text{O}}_{\text{2}}^{\bullet}$ → $\text{O}_{\text{2}}^{\bullet\text{-}}$ + H^+^ (82)

$\text{O}_{\text{2}}^{\bullet\text{-}}$ + H_2_O_2_ → ^1^O_2_ + OH^−^ (83)

Fe^2+^ + H_2_O_2_ → Fe^3+^ + ^•^OH + OH^−^ (84)

Fe^3+^ + H_2_O_2_ → Fe^2+^ + ${\text{H}\text{O}}_{\text{2}}^{\bullet}$ + H^+^ (85)

Fe^2+^ + ^•^OH → Fe^3+^ + OH^−^ (86)

S_2_$\text{O}_{\text{8}}^{\text{2-}}$ + Fe^2+^ → Fe^3+^ + $\text{SO}_{\text{4}}^{\text{2-}}$ + $\text{SO}_{\text{4}}^{\bullet\text{-}}$ (87)

Fe^2+^ + $\text{SO}_{\text{4}}^{\bullet\text{-}}$ → Fe^3+^ + $\text{SO}_{\text{4}}^{\text{2-}}$ (88)

S_2_$\text{O}_{\text{8}}^{\text{2-}}$ + ^•^OH → S_2_$\text{O}_{\text{8}}^{\text{•}\text{-}}$+ OH^−^ (89)

Fe^2+^ + $\text{SO}_{\text{4}}^{\text{2-}}$ $\leftrightarrow$ FeSO_4_ (90)

Fe^3+^ + $\text{SO}_{\text{4}}^{\text{2-}}$ $\leftrightarrow$ $\text{Fe}\text{SO}_{\text{4}}^{\text{+}}$ (91)

Catalysts$\underset{\to}{\text{photo}}$ *e^−^* + h^+^ (92)

h^+^ + OH^−^ → ^•^OH (93)

h^+^ + H_2_O → ^•^OH^*^ + H^+^ (94)

*e^−^* + O_2_ → $\text{O}_{\text{2}}^{\bullet\text{-}}$ (95)

**Supporting Figures**


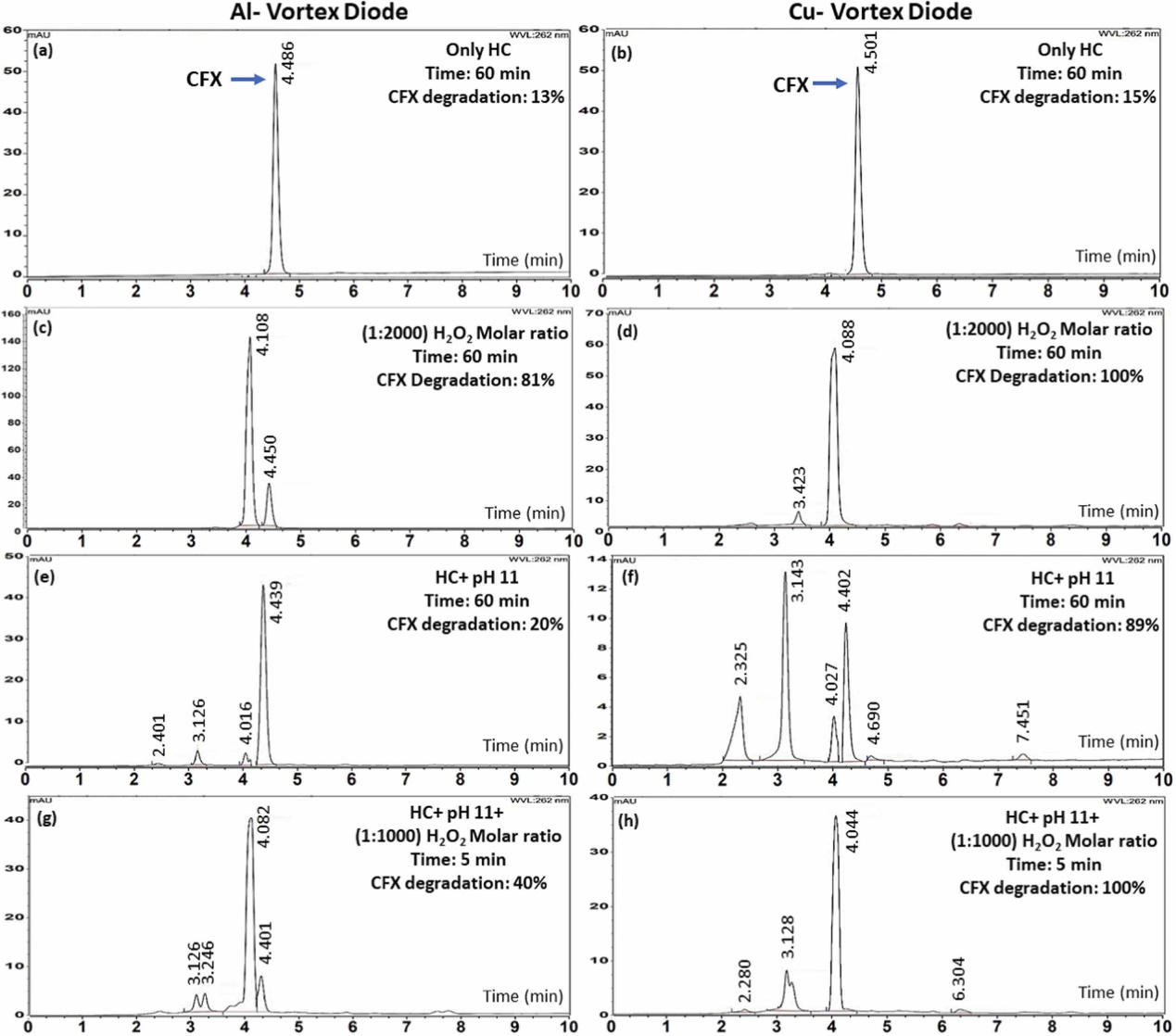


Fig. S1 HPLC analysis of CFX and by-products in various diode-based HC processes. Reprinted from *ref.* [1]. Copyright (2023), with permission from Elsevier.


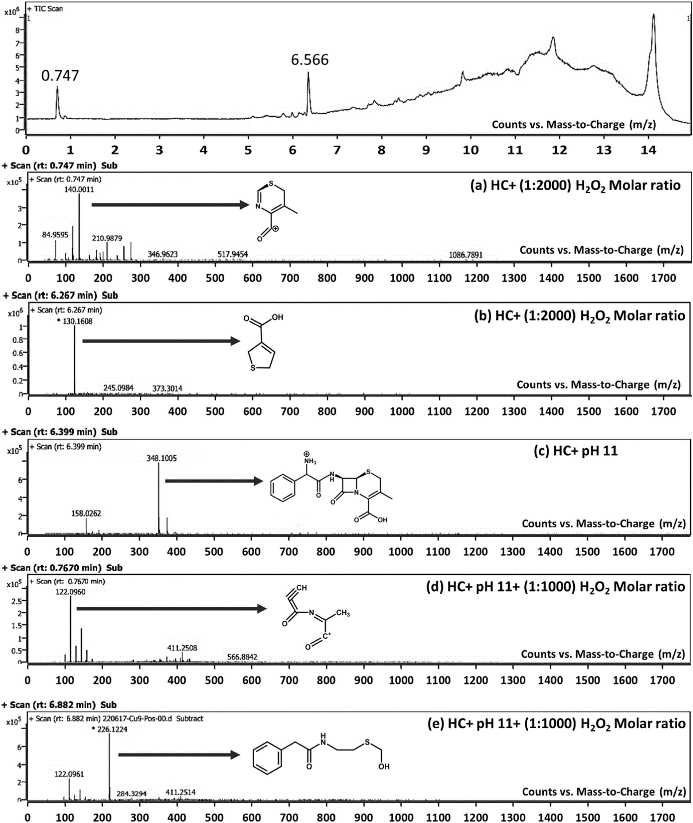


Fig. S2 LC-MS spectra for CFX degradation by-products for HC/H_2_O_2_ and hydrodynamic catalysis with pH adjustment. Reprinted from *ref.* [1]. Copyright (2023), with permission from Elsevier.


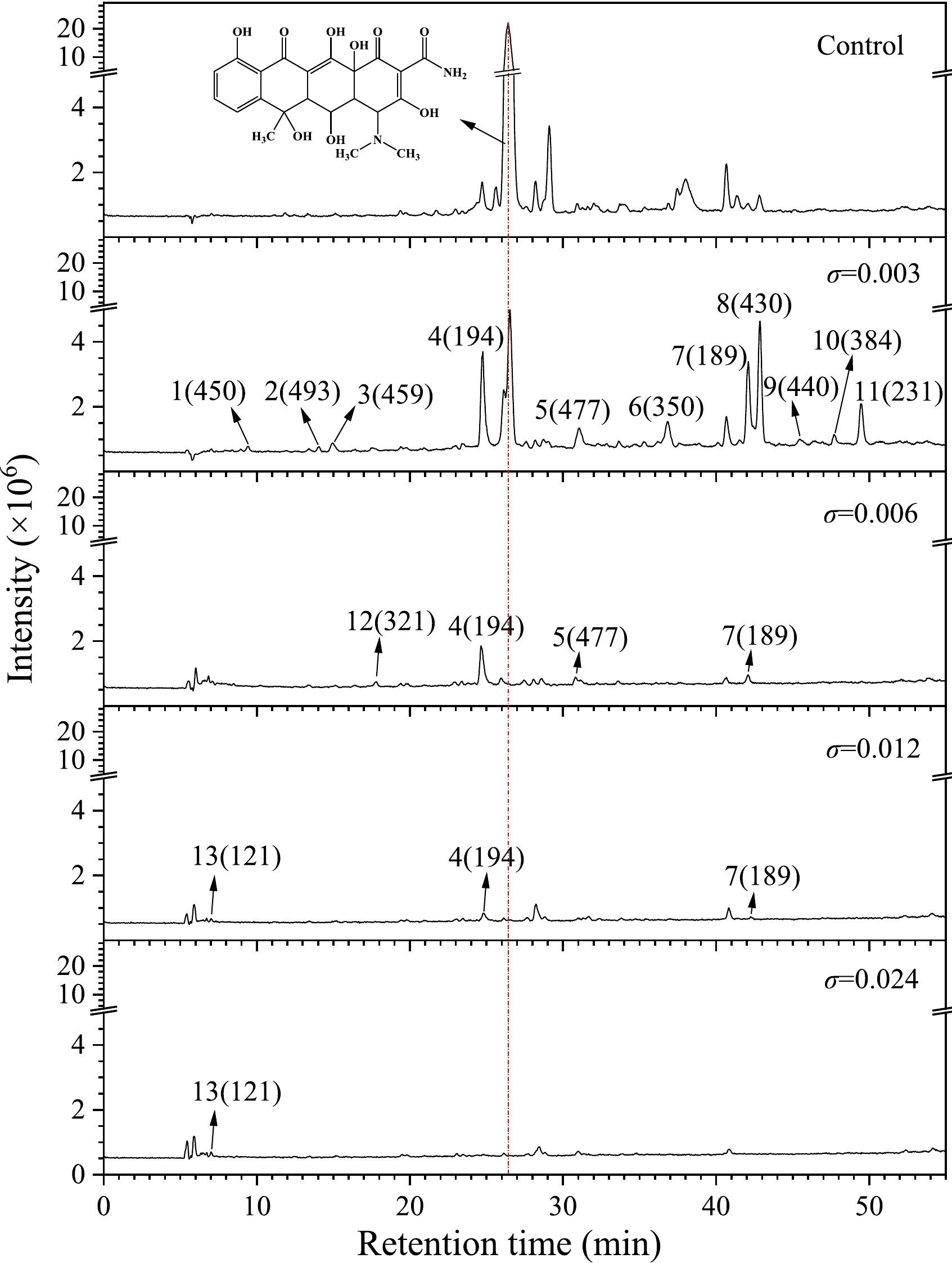


Fig. S3 LC-MS spectra for OTC degradation by-products for HC/O_3_ systems with different gas-liquid ratios (4 *μ*M and 3 s). Reprinted from *ref.* [2]. Copyright (2025), with permission from Elsevier.

**Supporting Tables**

Table S1 The chemical structures and bioactivities of common pharmaceuticals (Data were adopted from the Pubchem database).

| Pharmaceuticals | Chemical structures | p*K*_a_ | *LogK_OW_* | Bioactivities |
| --- | --- | --- | --- | --- |
| Atenolol |  | 9.54-9.60 | 0.16 | A cardioselective *β*_1_-adrenergic antagonist that lowers blood pressure and heart rate by selectively blocking the effects of adrenaline on the heart |
| Carbamazepine |  | 13.9 | 2.45 | A voltage-gated sodium channel blocker that stabilizes hyperexcited neuronal membranes and inhibits repetitive firing to manage epilepsy, trigeminal neuralgia, and bipolar disorder |
| Cephalexin |  | 2.53/  7.14 | 0.65 | A first-generation cephalosporin antibiotic that exerts bactericidal activity by binding to penicillin-binding proteins to inhibit the synthesis of the bacterial cell wall, leading to cell lysis and death |
| Ciprofloxacin |  | 6.16/  8.62 | 0.28 | A broad-spectrum fluoroquinolone antibiotic that exerts bactericidal activity by inhibiting DNA gyrase and topoisomerase IV, thereby disrupting bacterial DNA replication, transcription, and repair |
| Doxycycline |  | 3.09 | 0.63 | A second-generation tetracycline that primarily exerts bacteriostatic activity by reversibly binding to the 30S ribosomal subunit, effectively blocking the binding of aminoacyl-tRNA and inhibiting bacterial protein synthesis |
| Estradiol |  | 10.46 | 4.01 | The most potent endogenous estrogen that primarily functions as an estrogen receptor agonist, regulating the development of secondary sexual characteristics, the female reproductive cycle, and the maintenance of bone, cardiovascular, and neurological health |
| Neomycin |  | 12.9 | -3.7 | A potent aminoglycoside antibiotic that exerts rapid bactericidal activity by irreversibly binding to the 30S ribosomal subunit, which induces mRNA misreading and halts protein translocation, ultimately leading to the production of lethal, non-functional bacterial proteins |
| Metformin |  | 12.4 | -2.6 | A biguanide antihyperglycemic agent that primarily functions by activating AMP-activated protein kinase (AMPK) and inhibiting mitochondrial complex I, which together suppress hepatic gluconeogenesis and enhance peripheral insulin sensitivity |
| Oseltamivir phosphate |  | ~7.7 | ~1.1 | An oral prodrug that, following hepatic conversion to its active carboxylate form, functions as a potent neuraminidase inhibitor to block the cleavage of sialic acid residues, thereby preventing the release and spread of progeny influenza A and B virions from infected host cells |
| Oxytetracycline |  | 9.5 | -0.9 | A broad-spectrum bacteriostatic antibiotic that inhibits bacterial growth by reversibly binding to the 30S ribosomal subunit, which prevents the attachment of aminoacyl-tRNA to the mRNA-ribosome complex and effectively halts protein synthesis |
| Pefloxacin |  | 6.2/  7.5 | 0.27 | A second-generation fluoroquinolone that exerts bactericidal activity by inhibiting bacterial DNA gyrase and topoisomerase IV, thereby causing double-stranded DNA breaks and the cessation of DNA replication and transcription |
| Prazosin |  | 6.54 | 1.3 | A selective \alpha_{1}-adrenergic receptor antagonist that induces smooth muscle relaxation, primarily causing peripheral vasodilation to lower blood pressure and reducing internal urethral resistance to improve urinary flow |
| Sulfadiazine |  | 6.36 | -0.09 | A synthetic sulfonamide antibiotic that exerts bacteriostatic activity by competitively inhibiting the enzyme dihydropteroate synthase, thereby blocking the synthesis of bacterial folic acid and disrupting the production of essential DNA and RNA precursors |
| Tetracycline |  | 3.30 | -1.37 | A broad-spectrum bacteriostatic antibiotic that inhibits bacterial growth by reversibly binding to the 30S ribosomal subunit, which prevents the attachment of aminoacyl-tRNA to the ribosomal acceptor (A) site and halts the elongation of the polypeptide chain |

**References**

[1] J. Katiyar, R.G. Bhoi, V.K. Saharan, Improved degradation of oseltamivir phosphate, an antiviral drug, through hydrodynamic cavitation-based hybrid advanced oxidation processes: An insight into geometrical parameter optimization, Chem. Eng. Process. Process Intensif. 200 (2024) 109796. <https://doi.org/10.1016/j.cep.2024.109796>.

[2] Q. Wu, Doxycycline degradation via hydrodynamic cavitation combined photocatalysis: Optimization of geometric and operational parameters, Chem. Eng. Process. Process Intensif. 209 (2025) 110154. <https://doi.org/10.1016/j.cep.2025.110154>.
